# Meta-QTL for resistance to white mold in common bean

**DOI:** 10.1371/journal.pone.0171685

**Published:** 2017-02-15

**Authors:** Renato C. C. Vasconcellos, O. Blessing Oraguzie, Alvaro Soler, Haidar Arkwazee, James R. Myers, Juan J. Ferreira, Qijian Song, Phil McClean, Phillip N. Miklas

**Affiliations:** 1 Department of Biology, Federal University of Lavras, Lavras, Minas Gerais, Brazil; 2 Irrigated Agriculture Research and Extension Center, Washington State University, Prosser, Washington, United States of America; 3 USDA-ARS, Grain Legume Genetics and Physiology Research Unit, Prosser, Washington, United States of America; 4 Department of Horticulture, Oregon State University, Corvallis, Oregon, United States of America; 5 Área de Cultivos Hortofrutícolas y Forestales, Servicio Regional de Investigación y Desarrollo Agroalimentario (SERIDA), Villaviciosa, Asturias, Spain; 6 USDA-ARS, Soybean Genomics and Improvement Laboratory, Beltsville, Maryland, United States of America; 7 Department of Plant Sciences, North Dakota State University, Fargo, North Dakota, United Sates of America; University of Guelph, CANADA

## Abstract

White mold, caused by the fungus *Sclerotinia sclerotiorum* (Lib.) de Bary, is a major disease that limits common bean production and quality worldwide. The host-pathogen interaction is complex, with partial resistance in the host inherited as a quantitative trait with low to moderate heritability. Our objective was to identify meta-QTL conditioning partial resistance to white mold from individual QTL identified across multiple populations and environments. The physical positions for 37 individual QTL were identified across 14 recombinant inbred bi-parental populations (six new, three re-genotyped, and five from the literature). A meta-QTL analysis of the 37 QTL was conducted using the genetic linkage map of Stampede x Red Hawk population as the reference. The 37 QTL condensed into 17 named loci (12 previously named and five new) of which nine were defined as meta-QTL WM1.1, WM2.2, WM3.1, WM5.4, WM6.2, WM7.1, WM7.4, WM7.5, and WM8.3. The nine meta-QTL had confidence intervals ranging from 0.65 to 9.41 Mb. Candidate genes shown to express under *S*. *sclerotiorum* infection in other studies, including cell wall receptor kinase, *COI1*, ethylene responsive transcription factor, peroxidase, and MYB transcription factor, were found within the confidence interval for five of the meta-QTL. The nine meta-QTL are recommended as potential targets for MAS for partial resistance to white mold in common bean.

## Introduction

White mold, caused by the necrotrophic fungus *Sclerotinia sclerotiorum* (Lib.) de Bary, limits the yield potential and reduces the quality of seeds and pods of common bean (*Phaseolus vulgaris* L.). With favorable environmental conditions (prolonged wetness and moderate temperatures) and susceptible cultivars, white mold can cause up to 100% yield loss [1]. Integrated strategies to limit the advance of white mold in bean crops include crop rotation, fungicides, optimized irrigation and fertilizer to lessen biomass, and the use of less susceptible cultivars with erect growth habits that contribute to disease avoidance [2]. These control measures, however, may limit yield potential and increase production costs, so breeders continue to seek to develop cultivars with higher levels of field resistance to the fungus which incorporates both physiological resistance and disease avoidance.

Most known sources of physiological resistance to white mold are of Andean origin, or from a secondary gene pool, such as *Phaseolus coccineus* L. [3–8]. The lack of high levels of physiological resistance contributing to field resistance in commercial bean types combined with moderate to low heritability for the trait limits progress in breeding common bean cultivars with improved white mold resistance [1]. Moreover, it is difficult to discern physiological resistance mechanisms in the field, because of the additional influence of environment and disease avoidance traits [9].

Numerous QTL studies for white mold resistance and disease avoidance traits have been conducted in different common bean populations. Miklas et al. [2] used results from these studies to build a comparative map composed of 27 QTL for white mold resistance and 36 QTL for disease avoidance traits, shown to be widely distributed across the bean genome. It was difficult to comparatively map and discern physical positions for many of the QTL from these earlier white mold QTL studies due to the limited genetic coverage and the absence of shared framework markers.

The availability of an annotated reference genome sequence [10] and the development of an extensive set of genome-wide SNP markers [11] facilitated the development of denser linkage maps which enables the mapping of white mold resistance QTL to narrow intervals in common bean [12,13]. Hoyos-Villegas et al. [14] used SNP markers to validate and define the physical interval for the WM3.1 QTL for partial white mold resistance originally found [15] using a low density RAPD marker linkage map. The reference common bean genome sequence combined with sequence-based introgression mapping was used to locate the WM7.1 QTL for partial resistance to white mold to a 660 kb region on chromosome Pv07 and reduced the WM8.3 QTL interval to a 2.36 Mb region on chromosome Pv08 [16].

Further refinement of QTL intervals and the identification of more robust and reliable QTL, without incurring intensive resources, can be achieved using QTL meta-analysis [17]. The QTL meta-analysis is an approach to identify consensus QTL representing effects across different environments, genetic backgrounds, and related traits. This approach requires independent QTL for the same or related traits obtained from different populations, locations, or environmental conditions [17]. This method was useful for characterizing the genetic determinants of complex traits for a variety of crops, such as fiber quality, yield, drought tolerance and disease resistance in cotton (*Gossypium hirsutum*) [18], yield and anthesis silking in maize (*Zea mays*) [19], partial resistance to *Aphanomyces euteiches* in pea (*Pisum sativum*) [20], drought stress in rice (*Oryza sativa*) [21], ear emergence in wheat (*Triticum aestivum*) [22] and yield-associated traits in *Brassica juncea* [23]. Until now, the approach is largely unexplored for common bean.

Our objective was to leverage the new reference genome sequence and SNP marker arrays available for common bean for a QTL meta-analysis to identify consensus QTL for white mold resistance from multiple common bean populations and studies. Six recombinant inbred line (RIL) populations were populated with SNP markers and analyzed for WM QTL for the first time, three previously studied RIL populations were re-analyzed for WM QTL using denser SNP marker-based linkage maps, and WM QTL with discernible physical positions from five mapping populations from the literature [5,14,16] were used for the QTL meta-analysis.

## Materials and methods

### New RIL populations

Six new RIL mapping populations were generated to characterize partial resistance to white mold (Table 1). An advanced inbred backcross population, Orion//Orion/R31-83 (O83), consisting of 104 BC_1_F_5:7_ RILs was developed by single seed decent (SSD) from a BC_1_F_2_ population. The initial BC_1_F_2_ was developed by crossing the R31-83 partial resistance donor to the susceptible great northern ‘Orion’ with the resulting F_1_ backcrossed to Orion. R31-83 is a RIL from the Raven/I9365-31 (R31) black bean mapping population which possesses partial resistance to white mold [5].

**Table 1:** Characterization of 14 RIL populations used for meta QTL analysis for white mold resistance in common bean.

					Mapped markers		WM QTL identified	
RIL population	Parents (resistant parents in bold)(resistant parent in bold)		Putative resistance source	No. of RILs	This study	Previously published	This study	Previously published
**New populations**								
O83	Orion*2	**R31-83**	*P*. *coccineus*	104	342		1.1, 2.2, 7.6	
M25	Montrose	**I9365-25**	*P*. *coccineus*	130	564		5.4, 7.1, 7.5	
UI25	UI-537	**I9365-25**	*P*. *coccineus*	127	509		5.4, 6.2	
O12	Orion	**USPT-WM-12**	ICA Bunsi	158	315		1.2, 7.4, 9.3	
AO	**A195**	OSU6137	Andean	114	2532		1.1, 1.3, 3.3, 5.5, 7.4, 9.3	
GW	**G122**	**WMG9-04-20-3**	Andean & *P*. *coccineusP*. *coccineus*	80	1349		8.1	
**Re-genotyped populations**								
R31	Raven	**I9365-31**	*P*. *coccineus*	105	578	126	2.2, 5.4, 6.1, 7.5	2.2, 4.2, 5.3, 5.4, 6.1, 7.3, 8.4
AN	Aztec	**ND88-106-04**	ICA Bunsi	85	548	125	2.2, 3.1, 7.5	2.2, 3.1
XC	**Xana**	Cornell 49–242	Andean	113	762	294	1.1, 3.1, 6.2, 7.1, 7.4	1.1, 3.2, 6.1, 7.1, 7.4
**Existing populations**								
BV	Benton	**VA19**	Andean	79		48		2.2, 8.3
AP	**AN-37**	P02630	ICA Bunsi	94		447		3.1
PS02-011A	pinto*4	**G122**	Andean	38		10		7.1
PS02-029C	Matterhorn*4	**NY6020-4**	Andean	41		40		8.3
Z0725	**11A-29**	**29C-40**	Andean	79		introgressionmapping		7.1 and 8.3

The O83 RILs and the two parents were evaluated for reaction to white mold in the USDA-ARS greenhouses at Prosser, WA, using the straw test described by Petzoldt and Dickson [24]. The experimental design was a randomized complete block (RCBD) with six replications. The experiment was conducted during the winter months in 2013–2014. Temperatures were maintained at 21°C day and 16°C night and artificial high-intensity discharge (HID lamps) lights were utilized to maintain a 12 h day length. Two seeds of each RIL were planted in a 10 cm diameter square plastic pot containing Sunshine® brand SB40 professional growing mix (Sun Gro Horticulture, Agawam, Massachusetts) and 2.5 ml of Scott’s Osmocote® 14-14-14 slow-release fertilizer (A. M. Leonard, Inc Piqua, Ohio) applied at the time of planting. After emergence, pots were thinned to one plant and were watered as necessary for vigorous growth. The plants were inoculated with *S*. *sclerotinia* isolate T001.01 (5) approximately 28 d after planting and evaluated for disease severity at 7 and 11 d after inoculation using the 1–9 scale (24).

Field evaluation of O83 RILs and parents for white mold disease reaction was conducted in 2014 at the USDA-ARS Cropping Systems Research Farm at Paterson, WA (latitude N45.940446, longitude W119.489629, 135 masl), which has a history of uniform *S*. *sclerotiorum* disease in bean [1,15,25,26]. The soil is a Quincy sandy loam (mixed, mesic Typic Torripsamments). Field design included four-row plots with lines replicated twice and arranged in a randomized complete block design. The plots were 3 m in length with rows spaced 0.56 m apart. About 6.3 mm of water was applied daily by overhead center-pivot irrigation in the mid-afternoon from the first appearance of flowers until near physiological maturity. Six applications of nitrogen in the form of 20-0-0 NPK was foliar-applied weekly at a rate ~22.4 kg ha^-1^ to promote a full canopy favorable for white mold epidemics. Normal cultural practices for optimum growth were practiced. Reaction to white mold disease was estimated at physiological maturity using the scale from 1 = most resistant, to 9 = most susceptible, as previously described [25].

Two RIL populations ‘Montrose’/I9365-25 (M25) and ‘UI-537’/I9365-25 (U25) were generated using the I9365-25 pink bean germplasm line as a source of partial resistance to white mold. I9365-25 is derived from an interspecific cross between *P*. *vulgaris* x *P*. *coccineus* [27]. The partial resistance to white mold is considered to have been donated by the *P*. *coccineus* parent, a well described source of white mold resistance [2]. Montrose pinto and UI-537 pink bean are highly susceptible to white mold. Both have Type III prostrate growth habits (decumbent-indeterminate vine; [28]) which lack white mold disease avoidance characteristics and are prone to white mold infection. 130 and 127 F_5:7_ RILs were generated for the M25 and U25 populations, respectively. The RILs and parents were tested in the greenhouse with six replications and in the Paterson field nursery with three replications using a randomized complete block design following the same protocols described above for the O83 RIL population. The M25 population was tested in three row plots in the field in 2006, and the U25 population tested in one row plots in 2007. The susceptible pinto bean cultivar ‘Burke’ was planted every third row for the U25 field trial, such that each individual plot would have at least one common border.

158 F_5:7_ RILs were derived from the bi-parental cross Orion/USPT-WM-12 (O12). USPT-WM-12 is a pinto bean germplasm release [29] that exhibited good levels of partial resistance to white mold for many years in the greenhouse straw and field tests conducted by the Bean White Mold Nursery (BWMN) [30]. The RILs and parents were tested with six replications for the straw test and two replications for the Paterson farm field trial in 2014 using four row plots. Both trials used a randomized complete block design and followed the same protocols described above for the O83 trials.

Two snap bean populations, A195/OSU6137 (AO) with 114 F_5:7_ RILs and G122/WMG904-20-3 (GW) with 80 F_5:7_ RILs, were developed using single seed descent. The AO population was a cross between a white mold susceptible snap bean OSU6137 and a partially resistant dry bean A195 [31]. The GW population was a cross of resistant by resistant lines where G122 is a well characterized dry bean with partial resistance conferred by a QTL on Pv07, and WMG904-20-3 is a RIL derived from a backcross-inbred population used to introgress white mold resistance from the *P*. *coccineus* accession PI255956 into a snap bean background (the Bush Blue Lake cultivar OR91G was the recurrent parent). Previous work indicated that WMG904-20-3 had a QTL for resistance on Pv03 [32].

Both the AO and GW populations were evaluated for resistance to white mold in the straw test and field. A modified straw test conducted at the seedling stage was used. Four seeds from each family were planted in 10 cm pots in soilless media (Sungro Horticulture) supplemented with 3.5 g Osmocote 14-14-14 slow release fertilizer. Pots were replicated three times in a randomized complete block experimental design. For inoculation, actively growing mycelia from fungal sclerotia was produced on potato dextrose agar, and a plug of agar from the outer edge of the mycelia was taken to inoculate 7 to 10 day old seedlings. With the modified straw test method, the stem was cut 1–2 cm above the primary leaves, and a straw with two plugs of agar with fungal mycelia was placed on the decapitated stem. Seedlings were scored four days after inoculation using a 1–9 scale. Scoring was: 1 = No lesion, 3 = lesion reaching first node, 5 = lesion reaching half way between the first and cotyledon nodes, 7 = lesion reaching cotyledon node, 9 = seedling completely collapsed and dead.

Field trials were conducted on the OSU Vegetable Research Farm in Corvallis, OR (latitude N44.571209, longitude W123.243261 at 77 masl) on a Chehalis silty clay loam soil (Fine-silty, mixed, superactive, mesic Cumulic Ultic Haploxeroll) in 1.52 m plots in rows 0.76 m apart with a planting density of 50 seeds per plot. The field was fertilized with 168 kg ha^−1^ of 12-29-10 NPK banded in row at planting. Plot design was a randomized complete block with three replications. Both incidence (percent plants infected in a plot) and severity were measured. A scale of 1 to 9 was used to rate severity where 1 indicated no disease and 9 indicated that all plants were dead. The geometric mean for each plot was calculated based on incidence percentages and severity scores. Plots were watered using solid set sprinklers with approximately 2.5 cm applied on a weekly basis. Seed was pretreated with Captan (Bonide) pre-emergent fungicide to improve germination uniformity and reduce differences in stand between lines with different seed coat colors. The pre-emergent herbicide Dual (S-metolachlor, Syngenta) was applied at a rate of 1.1 kg ha^−1^ and seedings were treated with 2.33 L ha^−1^ of Sevin (1-naphthyl N-methylcarbamate, Bayer) insecticide for cucumber beetle control as needed. White mold inoculum came from naturally infested soils; the only environmental modification to increase *S*. *sclerotiorium* disease incidence and severity was from beginning bloom to physiological maturity to water the plots for 30 min every evening to increase the leaf wetness period.

### Re-genotyped RIL populations

Three RIL populations R31, AN, and XC (Table 1), previously characterized for QTL conditioning partial resistance to white mold, were re-analyzed for QTL using the same phenotypic data sets but with denser linkage maps generated by assaying 5398 SNPs from the BARCBean6K_3 bead chip [11] as described below.

The R31 (‘Raven’/I9365-31) population consisted of 105 F_5:7_ RILs that was originally populated with 126 RAPD and SSR markers [5]. Raven is black bean cultivar with Type II growth habit [28] and is highly susceptible to *S*. *sclerotiorum* [33]. I9365-31 is a black bean germplasm release from an interspecific cross between *P*. vulgaris*2/*P*. *coccineus* [27]. The R31 RILs were screened for reaction to white mold in the greenhouse straw test and the field [5].

The Aztec/ND88-106-04 (AN) RIL population with 85 F_5:7_ RILs was originally populated with 125 mostly RAPD markers and a few AFLP markers [15]. Aztec is a semi-upright pinto bean [34] susceptible to white mold. ND88–106–04, from the cross N85007/ICA Bunsi, is an upright navy bean breeding line with partial resistance to white mold derived from ICA Bunsi [35]. The 85 RILs and parents were screened for white mold reaction only in the field [36].

The linkage map for the Xana/Cornell 49–242 (XC) population, consisting of 113 F_7_ RILs and 294 markers [37], was originally constructed primarily using AFLP markers. This RIL population was tested against five different *S*. *sclerotiorum* isolates collected from northern Spain in the greenhouse straw test. Xana is a bean variety in the large white fabada market class developed at Servicio Regional de Investigación y Desarrollo Agroalimentario (SERIDA, Villaviciosa, Spain) with determinate, Type I growth habit. Cornell 49–242, is a very small-seeded black bean line, with indeterminate prostrate, Type III growth habit. The improved linkage map for XC integrating SNP markers from the BARCBean6K_3 BeadChip [11] was previously published [38]. The updated version of the XC genetic map has 11 linkage groups, covers 1394 cM, and includes 649 SNPs, as well as 113 INDEL, SSR, and SCAR markers.

O83, M25, U25, O12, R31, and AN were scored for traits associated with avoidance of white mold disease. The traits are: harvest maturity (days), canopy height (cm), green stem trait (1 to 5 scale), canopy porosity (1 to 5 scale), and lodging (1 to 9 scale). The role of these traits in disease avoidance are well described in previous studies [2,5,14,15]

### Existing mapping populations

The physical map locations of QTL conditioning partial resistance to white mold were also extracted from recent publications for use in the QTL meta-analysis. The physical position for the WM2.2 and WM8.3 QTL in the Benton/VA19 (BV) [5] genetic linkage map was estimated by a BLASTn analysis using the sequence-related amplified polymorphism (SRAP) markers (accessions FJ597978 to FJ597982 and FJ748892 to FJ748898) previously located in the vicinity of the QTL as the query and the reference genome sequence as the database. The physical location for the WM3.1 QTL in the AN-37/P02630 (AP) population was easily extracted from the SNP linkage map used to detect the QTL [14]. The physical positions for WM7.1 and WM8.3 QTL detected by sequence-based introgression mapping were obtained from multiple populations (PS02-011, PS02-029C and Z0725) [16].

### Genotyping with SNP markers

The O83, M25, U25, O12, R31, AN, and XC populations were genotyped using the BARCBean6K_3 BeadChip containing 5398 SNPs [11]. The SNP genotyping was conducted on the Illumina platform by following the Infinium HD Assay Ultra Protocol (Illumina Inc., San Diego, CA). The Infinium II assay protocol was used for genotyping and the genotyping was conducted following the procedures described previously [39].

The two snap bean RIL populations AO and GW were populated with SNP markers generated by genotype-by-sequencing (GBS) [40]. Leaf tissue was collected from a randomly selected single plant from each family grown in the field in the F_6_. Genomic DNA was isolated by OSU Center for Genome Research and Biocomputing (CGRB) from 50 mg of leaf tissue using Omega Biotek’s Mag Bind Plant DNA Plus kit. The process was completed on a KingFisher Flex extraction robot. The DNA was quantified using a fluorophore on a Synergy HT plate reader from Biotek, using the Quant-iT assay kit (Thermo Fisher).

For GBS, the plate of DNA was normalized using the epMotion 5075 liquid handling robot to 100 ng in 10 μl. Each sample was then digested in a 20 μl reaction using Apek I at 75°C for 2 h. Barcodes were then ligated to the cut ends using T4 Ligase at 22°C for 2 h. The library was then pooled (5 μl of each sample) into a single tube and a PCR clean-up was performed using QIAquick PCR Purification Kit (Qiagen) and eluted in 30 μl of elution buffer (EB, Qiagen). PCR was employed to amplify the library using 25 μl of 2X Phusion Taq Master Mix, with 3 μl of each Illumina primer at 10 μM each, along with 3 μl of the cleaned, pooled library, and 16 μl water to make a 50 μl reaction. Thermal parameters for the PCR were 1) 95°C 30 sec, 2) 95°C 10 sec, 3) 68°C 30 sec, 4) 72°C 30 sec (steps 2–4 repeated for 15 cycles), 5) 72°C 5 min, and 6) 4°C hold. The PCR reaction was cleaned using the QIAquick PCR Purification Kit and eluted in 30 μl EB. The library was then quantified using a Qubit reader (Thermo Fisher) and run on the Agilent 2200 Bioanalyzer to obtain fragment length distribution and find the median fragment length. The median fragment length and the concentration of the library were used to calculate a final dilution in EB at 10 nM which is optimal for hybridization to the flowcell on the cBot. The library was also run on qPCR to obtain a more accurate loading concentration for the cBot.

The library was then run on a HiSeq 3000 from Illumina on a 150bp paired-end flowcell. The data were packaged and ported through the Tassel 5 analysis pipeline [41]. Total number of SNPs obtained were 79,561 and 54,276 for GW and AO populations, respectively. After filtering to remove those with more than 20% missing values and less than 5% minor allele frequency, 25,772 and 15,034 SNPs remained for GW and AO, respectively. Thereafter, missing data were imputed in Beagle4.1 [42] using the default window size of 50000 SNPs and an overlap of 3000 SNPs.

### Genetic linkage map construction and QTL analysis

The polymorphic SNP markers were surveyed across the RILs of the respective populations (O83, M25, U25, O12, AO, GW, AN, R31, XC). Markers with >20% missing data or significant deviation from the expected Mendelian segregation ratios as determined by chi-square analysis were removed. Further filtering and linkage map construction was done using Joinmap 4.1 [43]. Linkage groups were established using a logarithm of the odds (LOD) threshold of 4.0 for significant pairwise marker linkages and a maximum genetic distance of 50 centimorgans (cM). Recombination frequencies were converted to (cM) using Haldane’s mapping function and maximum likelihood algorithm. Linkage groups were created based on known chromosome locations of the markers. Linkage group marker orientation was verified by comparing the final linkage map to the physical positions of the individual SNP markers on the bean reference genome [10]. Only a single marker from groups of markers that mapped at the same genetic position was included in the map to reduce redundancy.

Quantitative trait loci analysis was performed using the composite interval mapping (CIM) procedure [44] implemented in QGene 4.3 software [45]. To determine the LOD threshold, a permutation analysis was executed at 1000 permutations with an experimental error rate of 0.05. A scan interval of 1.0 cM was used, and the QTL support interval was defined as the flanking map intervals whose LOD score was <1 LOD value of the peak score. The closest marker at each putative QTL identified using interval mapping was selected as a cofactor. The *R*^2^ for the QTL was calculated for the peak QTL position. The physical boundary of the QTL was determined based on the positions of the closest markers flanking the support interval in the common bean reference genome sequence.

### QTL meta-analysis

The QTL meta-analysis was conducted following the approach of Goffinet and Gerber [17] using BioMercator v4.2 software [46,47]. A map file and a QTL file were prepared for the Biomercator v4.2 software according to its requirements. The linkage map for the Stampede x Red Hawk F_2_ population with 7040 SNPs markers with 1474 unique genetic positions [11] was used as the reference linkage map for the meta-QTL analysis. Genetic positions of SNPs in each population analyzed were converted to cM position in the Stampede x Red Hawk reference map based on physical position for the SNP in the reference genome [10]. Only the QTL with the highest LOD score was included for those QTL expressed in both the greenhouse straw test and field in the same population. For those QTL extracted from the literature which lacked a peak position, the QTL peak position was calculated as the average position directly between the two flanking markers which defined the QTL interval. The QTL from the different populations that mapped to the same physical positions with overlapping confidence intervals were assumed to be stable and were used by the software to integrate the QTL into a consensus meta-QTL. The model (of four generated) with the lowest Akaike information criterion (AIC) value was then used to predict the most probable meta-QTL position(s) for each linkage group.

## Results

**Fig 1:**
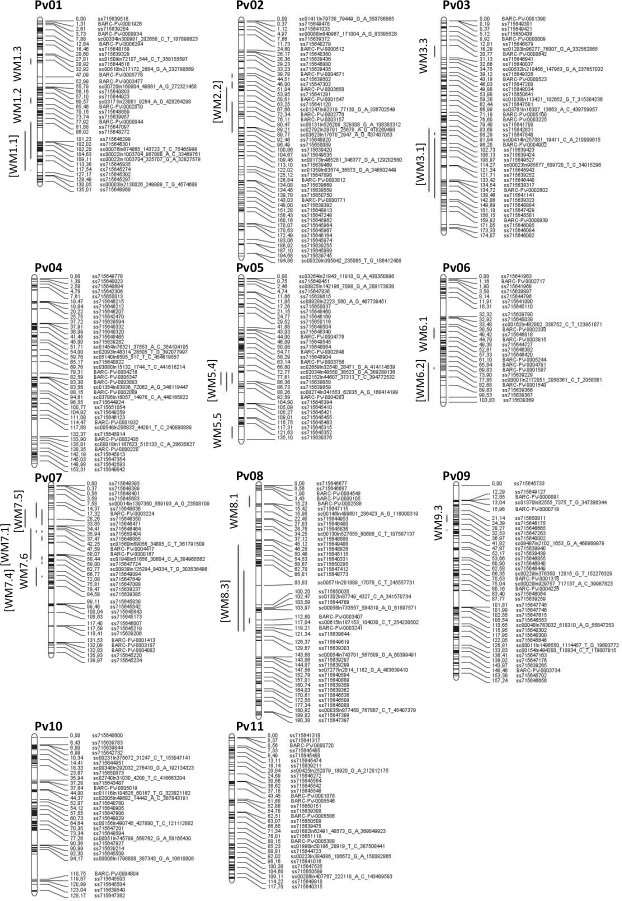
Representation of the individual QTL for resistance to white mold in the Stampede x Red Hawk common bean reference F_2_ genetic map based on physical mapping of the 37 QTL identified from 14 RIL populations. The nine meta-QTL are in brackets. Span of the QTL is represented by the length of the red line. The physical positions of the SNP markers across the 11 chromosomes can be found in the supplemental data of Song et al. [11].

The SNP-based linkage maps generated for the nine RIL populations (six new, three re-genotyped) are shown in Figs A to I in S1 File. 37 QTL (Table 2) conditioning partial resistance to white mold were identified from the 14 mapping populations. Of the 37 QTL, 20 were identified from the straw test evaluation, 13 from field trials, and four for both traits in the same population. Thirty-two of the 37 QTL derived from the resistant parent. The 37 QTL condensed into 17 named white mold QTL (12 existing and five new) (Fig 1). Nine of the 12 existing QTL were detected as meta-QTL WM1.1, WM2.2, WM3.1, WM5.4, WM6.2, WM7.1, WM7.4, WM7.5, and WM8.3 that will be reported in greater detail below. Most of the meta-QTL were defined by a narrower confidence interval than observed for any of the individual QTL from the 14 populations (Table 3). The exceptions were the WM7.1 and WM8.3 QTL from the sequence-based introgression mapping study [16] which had narrower confidence intervals (CI) than generated by the meta-QTL analysis. In addition the GBS SNP maps for AO and GW snap bean RIL populations detected individual QTL with quite narrow CI.

**Table 2:** Individual QTL for resistance to white mold in common bean identified across 14 RIL populations.

QTL	Population	Trait	Chromosome	QTL interval (cM)	QTL interval (Mb)	LOD	*R*^2^ (%)
WM1.1	(AO) A195/OSU6137	Field	Pv01	131.2–149.7	46.99–50.22	5.5	20.1
WM1.1	(O83) Orion//Orion/R31-83	ST^a^	Pv01	28.9–30.4	49.94–50.30	4.7	19.3
WM1.1	(XC) Xana/Cornell49242	ST	Pv01	108.9–129.1	45.15–49.31	6.0	26.1
WM1.2	(O12) Orion/USPT-WM-12	ST	Pv01	21.4–25.1	37.65–40.83	3.2	9.0
WM1.3	(AO) A195/OSU6137	ST	Pv01	69.4–79.1	13.73–17.31	3.6	13.7
WM2.2	(BV) Benton/VA19	ST/Field	Pv02	32.0–45.0	12.54–23.21	7.0	35.0
WM2.2	(O83) Orion//Orion/R31-83	ST	Pv02	17.4–18.7	3.11–3.57	9.2	34.7
WM2.2	(AN) Aztec/ND88–106–04	Field	Pv02	19.9–23.3	3.91–5.02	3.9	19.2
WM2.2	(R31) Raven/I9365-31	Field	Pv02	45.6–47.2	4.55–21.761	9.9	35.5
WM3.1	(AN) Aztec/ND88–106–04	Field	Pv03	47.7–52.1	36.10–38.27	4.4	13.7
WM3.1	(AP) AN-37/P02630	Field	Pv03	65.9–137.6	34.33–47.81	5.1	25.3
WM3.1	(XC) Xana/Cornell49242	ST	Pv03	69.3–102.9	39.43–48.32	3.6	16.7
WM3.3	(AO) A195/OSU6137	Field	Pv03	20.9–22.3	2.35–2.37	5.9	21.3
WM5.4	(M25) Montrose/I9365-25	ST/Field	Pv05	47.1–48.3	30.66–34.65	10.3	31.1
WM5.4	(U25) UI537/I9365-25	ST	Pv05	6.4–14.6	30.98–36.20	3.1	19.9
WM5.4	(R31) Raven/I9365-31	Field	Pv05	51.1–51.9	33.02–34.63	4.8	19.2
WM5.5	(AO) A195/OSU6137	ST	Pv05	124.9–126.2	39.83–40.01	3.9	14.7
WM6.1^b^	(R31) Raven/I9365-31	Field	Pv06	1.9–5.1	22.04–23.56	3.5	14.2
WM6.2	(XC) Xana/Cornell49242	ST	Pv06	120.1–136.1	28.28–31.74	2.8	13.0
WM6.2	(U25) UI537/I9365-25	ST	Pv06	33.1–34.8	28.90–29.06	3.1	19.8
WM7.5	(M25) Montrose/I9365-25	ST/Field	Pv07	21.4–27.2	1.81–2.67	3.2	10.8
WM7.5	(AN) Aztec/ND88–106–04	Field	Pv07	8.7–12.2	2.67–3.64	2.6	13.3
WM7.5	(R31) Raven/I9365-31	ST	Pv07	30.2–35.5	2.67–4.62	16.3	36.4
WM7.1	PS02-011	ST	Pv07	17.5–28.0	4.97–5.63	7.0	33.5
WM7.1	Z0725	ST	Pv07	4.5–8.0	4.97–5.63	8.1	37.4
WM7.1	(XC) Xana/Cornell49242	ST	Pv07	48.35–51.59	6.38–7.74	5.7	25.1
WM7.1	(M25) Montrose/I9365-25	Field	Pv07	36.1–50.1	4.62–15.77	4.2	13.8
WM7.4	(O12) Orion/USPT-WM-12	ST	Pv07	15.5–16.3	36.47–37.15	9.0	23.2
WM7.4	(AO) A195/OSU6137	ST	Pv07	94.0–96.7	41.80–42.41	3.6	13.7
WM7.4	(XC) Xana/Cornell49242	ST	Pv07	70.7–91.2	39.3–44.95	2.9	16.9
WM7.6	(O83) Orion//Orion/R31-83	ST	Pv07	51.6–53.1	9.33–11.94	6.6	26.1
WM8.1	(GW) G122/WMG904-20-3	ST/Field	Pv08	5.6–8.7	0.73–0.93	3.7	19.3
WM8.3	(BV) Benton/VA19	Field	Pv08	8.0–15.0	39.43–43.44	3.3	11.0
WM8.3	PS02-029C	ST	Pv08	8.5–14.0	4.97–46.71	2.7	27.6
WM8.3	Z0725	ST	Pv08	16.0–17.0	13.36–46.71	4.7	23.8
WM9.3	(AO) A195/OSU6137	Field	Pv09	17.0–20.6	9.97–11.51	4.7	17.4
WM9.3	(O12) Orion/USPT-WM-12	Field	Pv09	17.3–18.0	13.09–13.27	2.8	7.9

^a^ ST = straw test.

^b^ Underlined QTL derive from the susceptible parent and for G122/WMG904-20-3 which has both resistant parents, the WM8.1 QTL is derived from the WMG904-20-3 parent.

**Table 3:** Common bean white mold meta-QTL. The results are based on a meta-analysis of 37 QTL identified across 14 RIL populations and positioned relative to the Stampede x Red Hawk reference map [11].

			Interval position (Mb)					
Meta QTL	Trait	Chr	Start	Peak	End	R^2^ (%)	No. genes in interval	
WM1.1	ST^a^/Field	Pv01	49.57^b^	49.94	50.22	22^c^		76
WM2.2	ST/Field	Pv02	3.57	3.86	5.24	31		190
WM3.1	ST/Field	Pv03	34.96	36.22	39.49	19		341
WM5.4	ST/Field	Pv05	30.65	33.63	34.78	23		216
WM6.2	ST	Pv06	28.47	29.18	30.08	16		156
WM7.5	ST/Field	Pv07	1.81	3.30	4.06	20		258
WM7.1	ST/Field	Pv07	4.59	5.42	6.38	27		149
WM7.4	ST	Pv07	34.40	36.79	37.87	18		95
WM8.3	ST/Field	Pv08	37.32	44.70	46.73	21		215

^a^ ST = Straw test.

^b^ The actual SNPs for the start, peak, and ends of each meta-QTL are described in S1 Table.

^c^ Averaged R2 (PVE–phenotypic variation explained) of QTL within the same meta-QTL cluster.

### Chromosome Pv01

Two QTL were previously identified on Pv01. WM1.1 conditioned field resistance in the A55/G122 (AG) [25] RIL population likely due to disease avoidance conditioned by the *fin* gene for determinant bush growth habit. A QTL in a similar location was observed in the XC RIL population originally [48], and after the remapping conducted herein (Table 2, Fig J in S1 File), but it conditioned partial physiological resistance in the greenhouse straw test. The WM1.1 QTL was also observed in the O83 (Fig K in S1 File) and AO (Fig L in S1 File) populations and similar to XC conditioned partial physiological resistance in the greenhouse straw test. The consensus meta-QTL obtained from the QTL found across these populations was physically mapped to a 650 kb interval between 49.57 to 50.22 Mb. This meta-QTL is WM1.1 and is more representative of the QTL from XC, AO, and O83 than the AG QTL defined by the *fin* locus near 45 Mb [49] (Fig 1).

The second QTL, WM1.2, was first discovered in the G122/CO72548 (GC) RIL mapping population [50], and conditioned partial resistance in the greenhouse straw test. The QTL was located 20 cM from the *fin* locus in GC population, but the orientation to *fin* was uncertain due to the lack of framework markers in the region. If the distal orientation for WM1.2 from GC, which is nearest the BMd10 (49.04 Mb) and Bng (49.72 Mb) markers in the consensus map [5], is considered as the most likely position for this QTL, then it is equivalent to the WM1.1 defined by the meta QTL-analysis.

A single QTL WM1.2 for straw test resistance was observed in the O12 population at the 37.65–40.83 Mb interval (Fig M in S1 File). This location better coincides with the proximal orientation for the WM1.2 QTL from the GC population and occurs outside the CI for the WM1.1 QTL. A new QTL WM1.3 conditioning straw test resistance was identified in AO population in a distinct location (17.30–13.81 Mb) from the previous named QTL on Pv01.

A few candidate genes within the WM1.1 meta-QTL confidence interval (49.57 to 50.22 Mb) are worth mentioning. The candidate gene Phvul.001G236600 (49.70 Mb) is homologous to a gene in *Arabdopsis thaliana* (AT5G02070) and two genes, BnaCnng03730D and BnaA10g27400D, from *Brassica napus* that were up-regulated under *S*. *sclerotiorum* infection only in the resistant *B*. *napus* line [51]. This gene encodes a wall-associated receptor kinase protein that physically links cell wall to the plasma membrane. Wall receptor kinases can be involved in recognizing cell wall changes during pathogen entry leading to initiation of a signal transduction cascade [52–54].

Another candidate gene within WM1.1 is Phvul.001G240400 (50.03 Mb) which is homologous with a gene in *A*. *thaliana* that encodes a coronatine-insensitive protein 1 (*COI1*) which is a jasmonate receptor known to facilitate plant defense responses against necrotrophic pathogens. Loss of function mutant plants of *A*. *thaliana* that didn`t expressed *COI1* showed increased susceptibility to *S*. *sclerotiorum* and other necrotrophic pathogens [55,56].

### Chromosome Pv02

The PV02 WM2.2 QTL was reported in at least six populations and was associated with disease response in both greenhouse and field trials. WM2.2 QTL, reported previously for the R31 [5] and AN [15] populations, was detected again using the denser SNP linkage maps (Fig N in S1 File). A physical location for WM2.2 from BV was deduced by locating the flanking SRAP marker sequences on the reference genome. Lastly, WM2.2 was detected in the O83 backcross population. The resistance from R31-83 derives from I9365-31 which supports identification of the same QTL in both R31 and O83 populations. The meta-QTL WM2.2 maps to a 1.67 Mb region between 3.57 and 5.24 Mb.

Chalcone synthase *ChS* (3.76 Mb), pathogenesis-related protein *PvPR-2* (36.94 Mb) [57], and polygalacturonase-inhibiting protein family *Pgip* (36.04–36.20 Mb) [58] are all genes on Pv02 implicated in plant defense against fungal pathogens. The putative role of these genes in white mold resistance was previously discussed [15,59,60], but only *ChS* is located within the confidence interval for the WM2.2 meta-QTL.

Two tandem candidate genes, Phvul.002G055700 and Phvul.002G055800 located at positions 5.67 Mb and 5.69 Mb just outside the WM2.2 meta-QTL confidence interval are worth mentioning. These ethylene-responsive transcription factors are associated with the large bursts of ethylene that occur during the early stages of pathogen infection and can induce defense- and programed cell death-related genes [56]. Furthermore, the induction of ethylene responsive transcription factors in presence of *S*. *sclerotiorum* has been reported in *B*. *napus* [56,61–63].

### Chromosome Pv03

The WM3.1 QTL, previously reported [15] in Pv03 to condition field resistance in the AN RIL population, was located between markers BMd1 (27.04 Mb) and Bng165 (43.00 Mb) in the consensus map [5]. Hoyos-Villegas et al. [14] recently validated WM3.1 in the AN-37/P02630 (AP) population. Note that AN-37 is a RIL from the AN RIL population that was released as the white mold resistance pinto bean germplasm line USPT-WM-1 [64]. After re-genotyping the AN population with SNPs, WM3.1 was detected again and overlapped the QTL detected in the AP population. QTL WM3.2 from XC conditioning straw test resistance, upon remapping in this study, occurred within the WM3.1 interval observed for the AP population. Collectively, the WM3.1 QTL from the three populations detected a meta-QTL between 34.96 and 39.49 Mb. The QTL detected in AO population (2.35–2.37 Mb) has a physical location that is distinct from the WM 3.1 CI, so it was named WM3.3.

Although WM3.1 is associated with disease avoidance traits and the CI is seemingly too wide to effectively parse through hundreds of genes, there is one candidate gene, Phvul.003G164600 (37.29 Mb), found to encode a peroxidase that should be mentioned. Peroxidases are ubiquitous enzymes present in plants, animals and microorganisms. In plants, peroxidases are involved in physiological activities such as growth and development, through formation of lignin or oxidative carboxylation of indole-3-acetic acid (IAA) required for auxin metabolism [65,66]. Additionally, peroxidases play a crucial role in the generation of reactive oxygen species (ROS) during biotic and abiotic stress. Upon pathogen attack, several peroxidase genes are induced leading to over accumulation of ROS in plants which in turn causes oxidation of membranes and proteins, nucleic acid damage, onset of hypersensitive response and downstream signaling pathways, that could restrict pathogen spread from the site of infection [65]. Furthermore, up regulation of peroxidase genes lead to cell wall fortification through formation of lignin and suberin which reinforce the plant cell wall [67]. Leite et al. [68] showed that common bean lines that were more resistant to *S*. *sclerotiorum* also had a more intense activity of peroxidases.

### Chromosome Pv04

Although a meta-QTL was not identified Soule et al. [5] identified WM4.2 in the R31 RIL population, which we revisited with more markers and this QTL was not considered significant. Also, Park et al. [69] detected a QTL expressed in the field and in the greenhouse near the *RbcS* gene for the PC-50/XAN-159 (PX) population.

### Chromosome Pv05

Meta-QTL WM5.4 (30.65–34.78 Mb), was detected for QTL conditioning partial resistance in the greenhouse and field identified across the M25, U25 and R31 populations (Figs O to Q in S1 File). Both resistant parents for these three populations, I9365-25 and I9365-31, derive from an interspecific population between *P*. *vulgaris* and *P*. *coccineus* suggesting that this meta-QTL may derive from the *P*. *coccineus* gene pool. Soule et al. [5] identified two QTL, WM5.3 and WM5.4, very close together for the R31 RIL population, but upon mapping with more markers herein only the one QTL WM5.4 was observed (Fig Q in S1 File).

The WM5.1 QTL in PX population is near RFLP D1157 (36.95 Mb) in the consensus map [5] which is outside the CI for WM5.4 and between the QTL (39.83–40.00 Mb) conditioning straw test resistance identified in AO. This QTL in AO is named WM5.5.

A candidate gene for meta-QTL WM5.4, Phvul.005G115500 (33.41 Mb), was homologous with a gene in Arabidopsis which encodes a MYB domain protein. In *A*. *thaliana*, MYB transcription factors play a vital role in several diverse functions such as regulatory networks that control development, metabolism and responses to biotic and abiotic stresses [70]. Most noteworthy, MYB transcription factors were differentially expressed in soybean and *B*. *napus* in the presence of *S*. *sclerotiorum* [62,63,71].

### Chromosome Pv06

The previously reported Pv06 WM6.1 QTL in the consensus map [2] was loosely flanked by the SSR markers BMd12 (17.99 Mb) and PV-at004 (26.86 Mb) for the BN [26], R31 [5] and XC [48] populations. Remapping efforts in R31 and XC depicted close but separate QTL, the original WM6.1 for field resistance in R31 and the WM6.2 for straw test resistance in XC (Fig J in S1 File). The WM6.2 QTL for straw test resistance was also detected in the U25 population (Fig P in S1 File). However, all three of the individual QTL on Pv06 derive from the susceptible parents: Raven, Cornell 49–242 and UI-537. The WM6.1 QTL conditioning field resistance could be related to avoidance in the otherwise white mold susceptible parent Raven which has upright Type II growth habit.

The WM6.2 meta-QTL (28.47–30.08 Mb) obtained from the WM6.2 QTL for XC and U25 RIL populations has two obvious tandem candidate genes [Phvul.006G183100 (29.21 Mb) and Phvul.006G183200 (29.23 Mb)] that encode an ethylene-responsive transcription factor similar to the candidate genes near WM2.2.

### Chromosome Pv07

Miklas et al. [2] placed five QTL WM7.1 to 7.5 on Pv07. In this study, three of those five QTL, WM7.1, WM7.4, and WM7.5 were defined as meta-QTL (Table 3, Fig 1). The WM7.1 QTL expressed in the straw test and field and located near the Phaseolin seed protein locus (*Phs*– 4.95 Mb) was first reported in the A55/G122 [25] and PC-50/XAN-159 [68] RIL populations. Subsequently, Pérez-Vega et al. [48] observed WM7.1 and it was detected again with the SNP map for the XC population (Fig J in S1 File). This QTL is thought to derive from Andean bean (eg. G122, PC-50, and Xana) so it was surprising to detect it in the M25 RIL population (Fig O in S1 File) which derives from a Middle American pinto ‘Montrose’ crossed with a pink bean from an interspecific *Pv* x *Pc* population.

Miklas [72] used marker-assisted backcrossing to move WM7.1 from the Andean parent G122 into a pinto bean background. The resulting near-isogenic inbred pinto bean population PS02-011A with WM7.1, and a separate RIL population, Z0725, combined WM7.1 and WM8.3 QTL in a pinto bean background. These populations were used to fine map WM7.1 to a narrow 660 kb interval (4.97–5.63 Mb [16]. The WM7.1 meta-QTL detected in this study (Fig 1) has a slightly broader CI (4.59–6.38). See [16] for candidate genes within the region.

The QTL WM7.3 was previously mapped in the R31 RIL population [5], near the Bng204 marker (37.40 Mb), although when we revisited this population with more markers this QTL was not detected. Pérez-Vega et al. [48] identified WM7.4 QTL in the XC population, which is adjacent to WM7.3 in the comparative map [2,5]. That QTL was detected again with the denser SNP marker linkage map (Fig J in S1 File). A WM7.4 QTL conditioning partial resistance in the straw test was also detected in the AO (Fig L in S1 File) and O12 (Fig M in S1 File) populations. The WM7.4 meta-QTL has a 3.47 Mb interval between 34.40–37.87 Mb (Fig 1) but no obvious candidate genes were detected for this interval.

The third meta-QTL, WM7.5, has a 2.25 Mb CI (1.8–4.06) and is proximal but very close to WM7.1. This WM7.5 QTL was detected in the straw test and field in the AN, M25, and R31 populations. Note that WM7.5 was not detected with the original linkage maps [5,15] for AN (Fig N in S1 File) and R31 (Fig Q in S1 File). WM7.5 in M25 was detected in both the straw test and field (Fig O in S1 File). This QTL does not have an obvious Andean origin which further distinguishes it from WM7.1. WM7.5 was first reported as derived from the plant introduction landrace in the Tacana/PI 313850 RIL population [73]. No candidate genes were apparent for the WM7.5 meta-QTL.

The QTL WM7.6 conferring partial resistance in the straw test in O83 RIL population (Fig K in S1 File) has a distinct physical position (9.33–11.94 Mb) from all the other previously identified QTL on Pv07 and, pending validation, may represent a new QTL.

### Chromosome Pv08

The previously reported WM8.1 QTL in the PX [69] and GC [50] populations is flanked by marker BMd25 (0.93 Mb) and was reported to associate with disease avoidance traits in the PX population. The WM8.1 QTL, detected in the GW RIL population (0.64–0.73 Mb) (Fig R in S1 File) was expressed in the field and straw test. Note that the WM8.4 QTL in the original R31 linkage map [5] was not detected in the SNP map for R31.

The WM8.3 QTL was previously reported in BN [26], GC [50], and BV [5] populations. The WM8.3 QTL was transferred from the snap bean NY6020-4 into great northern bean market class using MAS [72]. Mamidi et al. [16] used the near-isogenic great northern inbred line population PS02-029C and a RIL population Z0725 from that study [72] to fine map the WM8.3 QTL region. They found wide confidence intervals for WM8.3 in PS02-029C (4.97–46.71 Mb) and Z0725 (13.36–46.71 Mb) populations using QTL interval mapping with Indel markers because the QTL spanned the ‘recombination poor’ heterochromatic region. We used these CI for the meta-QTL analysis.

Using a sequence based introgression mapping approach [16] the WM8.3 interval was significantly narrowed to 2.36 Mb (44.35–46.71 Mb). The WM8.3 meta-QTL has a slightly broader CI (40.62–46.60 Mb), but nonetheless it validates the narrowed location for the WM8.3 QTL [16]. Mamidi et al. [16] suggested Phvul.008G173600 (45.62 Mb) as a candidate gene for WM8.3. This gene is a common bean receptor-like protein (RLP) which can recognize external signals and transmit a response signal. Some *Arabidopsis* RLPs are involved in disease defense [74], and one RLP, *AtRLP30*, is known to mediate resistance to *Sclerotinia* [75].

### Chromosome Pv09

WM tolerance QTL were detected in the AO (straw test) and O12 (field) RIL populations (Figs L and M in S1 File) that physically map next to each other but do not overlap (Table 2). Due to the proximity of the QTL they will be given the same name for now–WM9.3. This QTL is physically positioned between WM 9.1 QTL in GC [50] near SSR marker BM184 (1.71 Mb) and WM9.2 in Tacana/PI 318695 [73] near RFLP marker Bng24 (26.40 Mb), as depicted in the comparative map [2].

No QTL were detected on Pv10 or Pv11 in the 14 populations in this study; however, others detected QTL on these chromosomes. Lara et al. [76] reported a QTL on Pv10 near the BM212 marker (43.2 M) using a Bayesian approach for the QTL identification. Mkwaila et al. [73] and Park et al. [69] identified a QTL WM11.1 [2] in the same proximal region on Pv11.

### Disease avoidance traits

A QTL for plant height (2.8 LOD) and canopy porosity (6.0 LOD) co-located with the WM2.2 QTL in AN (Fig N in S1 File) and R31 (Fig Q in S1 File) populations, respectively. There were QTL for canopy porosity (4.6 LOD), harvest maturity (5.5 LOD) and stay green stem trait (5.4 LOD) co-located with WM3.1 in the AN (Fig N in S1 File), and harvest maturity (LOD = 7.2) with WM3.1 in the AP [14] populations. The WM5.4 QTL co-located with QTL for canopy height (5.4 LOD) and porosity (6.0 LOD) in R31 population (Figs G and Q in S1 File), and harvest maturity (4.6 LOD) in M25 population (Fig O in S1 File). Pérez-Vega et al. [48] observed that increased plant height QTL measured in the greenhouse co-located with QTL conditioning reduced disease severity in the straw test reaction.

## Discussion

In this study, 37 individual QTL were detected across the 14 RIL populations. The 37 QTL coalesced into 17 named WM QTL, 12 previous and five new (WM1.3, WM5.5, WM6.2, WM7.6 and WM9.3). There were 27 white mold QTL comparatively mapped [2,5] from earlier studies that could not be incorporated in this meta-QTL analysis because they lacked physical genomic positions. Nonetheless, nine meta-QTL conditioning partial resistance to white mold were defined from the 37 individual QTL detected across the 14 populations examined in this study.

The AN and AP populations were only tested in the field because they lacked straw test resistance, XC population was only tested in the straw test, and only straw test results were reported for PS02-011A, PS02-029C, and Z0725 populations [16]. The other eight populations were tested in both greenhouse and field environments. Overall, there was a balance between individual QTL detected by the straw test (20) and field trials (13), but only four QTL were expressed in both environments, which supports the moderate to low correlations observed between greenhouse and field evaluations in some studies.

Disease avoidance traits expressed in the field are unlikely to be detected by the straw test which contributes to a lack of correlation between these tests. Those individual QTL expressed in both the straw test and field: WM2.2 (BV), WM5.4 (M25), WM7.5 (M25) and WM8.1 (GW), were generally observed in populations with moderate correlations between straw tests and field results (0.25*, BV; 0.26**, M25; 0.51***, GW) and not observed in populations that lacked significant correlations (-0.09 O83; 0.07 O12; 0.13 AO; -0.03 U25). Physiological resistance in the field is critical when avoidance traits are overcome by moderate to severe disease pressure; therefore, the goal of many breeding programs has been to combine physiological resistance (detected by the greenhouse straw test) with disease avoidance traits to enhance overall field resistance.

Of the nine meta-QTL detected, six (WM2.2, WM3.1, WM5.4, WM7.5, WM7.1 and WM8.3) were based on a combination of individual QTL from different populations expressed in the greenhouse, field, or both environments. Within these six meta-QTL, are there separate genes that are associated with greenhouse and field resistance? Upon examination of harvest maturity, canopy porosity, canopy height, and lodging data, only meta-QTL WM2.2, WM3.1, and WM5.4 were clearly associated with disease avoidance traits. So although the putative candidate genes described for the meta-QTL all condition a physiological disease resistance response process, investigation of other candidate genes with a putative role in disease avoidance traits is warranted.

There are three general sources of resistance represented by the 14 populations (Table 1): the Andean sources G122 (GW, PS02-011A and Z0725), Xana (XC), A195 (AO), NY6020-4 (PS02-029C and Z0725) and VA19 (BV); the ICA Bunsi source (AN, AP, O12); and the putative *P*. *coccineus* sources I9365-25, I9365-31 and WMG904-20-3 (R31, M25, U25, O83, GW). The WM5.4 and WM8.3 meta-QTL have individual QTL from one general source, WM 2.2 has QTL from all three general sources, and the other six meta-QTL have QTL from two general sources. This interpretation suggests that the three general sources of resistance have some genes in common that influence partial resistance to white mold, but perhaps possess different alleles for some of the loci. If different alleles conferring partial resistance exist, then it would be useful to determine which alleles at the common loci condition the highest level of resistance and/or have reduced linkage drag from deleterious genes located nearby. This might be done by using marker-assisted backcrossing to develop near-isogenic lines for the different alleles in a common recurrent parent. Meanwhile, from a breeding perspective it would be useful to choose general resistance sources most adapted to the genetic background you are trying to improve. For instance to improve resistance in an Andean market class one could choose combinations amongst meta-QTL WM1.1, WM2.2, WM3.1, WM7.1, WM7.4, WM8.3, and WM9.3 from the Andean sources (Table 1). Singh et al. [77] used four sources of Andean resistance A195, G122, MO162 and VA19 to develop an Andean line SE154 with superior level of physiological resistance to white mold in the straw test. To improve resistance in pinto bean, one may choose combinations amongst meta-QTL WM1.1, WM2.2, WM3.1, WM5.4, WM7.4, WM7.5, and WM9.3 from the ICA Bunsi and *P*. *coccineus* sources.

Similarly, it is important to combine general sources of resistance to generate novel combinations of QTL to achieve even higher levels of partial resistance to white mold. Singh et al. 77] achieved high levels of resistance in the straw test with the release of SE153 (syn PRP153; [78]) and the development of SE155 pinto breeding lines from multi-parent crosses that included common bean parents representing all three general sources of resistance: Andean, ICA Bunsi, and *P*. *coccineus* [7,79]. Viteri et al. [80] used linked markers to show that SE155-9 possessed QTL WM2.2, WM7.1 and WM8.3. Crossing SE155-9 breeding line with lines possessing other meta-QTL (WM3.1, WM5.4, WM7.5) expressed in the field, and then selecting among progeny for agronomic performance and resistance under severe disease pressure, may be a worthwhile strategy toward developing commercial cultivars with improved field resistance. An inherent problem with developing lines with improved resistance to white mold from wide crosses is that they generally lack acceptable agronomic performance and commercially acceptable ‘market class’ quality seed.

Six new RIL populations were mapped in this study. The O83 RIL population was developed to verify WM7.3 and introgress it into susceptible great northern bean. The R31-83 parent of O83 population is a RIL with straw test resistance from the R31 population. The WM7.3 QTL was originally identified in the R31 population with a large straw test effect (51 PVE-percent phenotypic variation explained) [5]. Validation of WM7.3, though, failed as it was not detected in the R31 (re-genotyped) or O83 populations. Instead, the WM7.5 was detected as in R31 as a large effect straw test QTL (36 PVE) at the proximal end of the chromosome (2.67–4.62 Mb). For O83, the large effect straw test QTL (26 PVE) on Pv07 was derived from the susceptible parent Orion. It mapped (9.33–11.44 Mb) adjacent WM7.5 but to a different location and was named a new QTL–WM7.6. Although, our effort to transfer and validate the WM7.3 QTL failed, a narrower CI for the WM7.5 QTL from R31, will facilitate another attempt to transfer this QTL with similar strong straw test effect into susceptible great northern or pinto bean using I9365-31 instead of a RIL as the source of the QTL. The importance of WM7.5 is further supported by its identification as a meta-QTL due to detection in the straw test in the M25 population.

The I9365-25 parent of M25 is similarly derived from an interspecific *P*. *vulgaris* x *P*. *coccineus* population as is the I9365-31 parent in the R31 population. This parentage supports the conclusion that WM5.4 and WM7.5, detected in both populations, are derived from *P*. *coccineus*. The U25 population also has I9365-25 as a parent, but only one QTL WM5.4 is found in both U25 and M25 populations. The detection of only a single common QTL between mapping populations with a common resistant parent attests to the complexity of the partial resistance response to white mold and reinforces the continuous need to validate QTL that are identified in only one population or environment. The WM7.1 QTL detected in M25 RIL population does not necessarily fit within the Andean origin for this QTL, but it has a relatively wide CI (4.62–15.77 Mb) which upon fine mapping may shift position of the QTL into the CI for WM7.5 or WM7.6. The proximal end of Pv07 in general is an important genomic region for white mold resistance because meta-QTL WM7.1 (4.59–6.38 Mb) and WM7.5 (1.81–4.06 Mb) are closely linked. The tight linkage in repulsion represents a challenge to breeders trying to combine these QTL, a challenge that is more manageable with robust markers.

The O12 RIL population was developed to characterize the consistent high level of partial resistance in the straw test exhibited by the USPT-WM-12 pinto germplasm line [30]. USPT-WM-12 was bred for white mold resistance based entirely on selection under disease pressure in the field [29]. The field resistance ultimately is thought to derive from ICA Bunsi which does not exhibit straw test resistance. So the discovery of partial greenhouse resistance in USPT-WM-12 was unexpected and could be important for breeders to select going forward. The findings from this study indicate the partial resistance of USPT-WM-12 in the straw test now has a genetic basis due in part to QTL WM1.2 and the meta-QTL WM7.4.

The AO population represents a cross to characterize the white mold resistance of A195 in a snap bean background. A195 is partially resistant to white mold in both the straw and field tests. Accordingly, six QTL were detected in AO, three in the straw test (WM1.3, WM5.5, and WM7.4), and three different QTL in the field (WM1.1, WM3.2, and WM9.3). Only two, WM1.1 and WM7.4, were observed to be meta-QTL. Viteri et al. [80] determined two complementary dominant genes condition the resistance from A195 in the straw test in a cross to susceptible ‘Othello’, and no segregation or a single dominant gene (depending on isolate) was observed when crossed to partially resistant G122. Viteri et al. [80] did not map the resistance loci that they identified, and our results are generally compatible.

The GW population was developed from a cross of partially resistant G122 previously reported to have QTL on Pv07 and Pv08 [25,50], and partially resistant WMG904-20-3 with possible QTL on Pv03 [32]. Only the QTL WM8.1 was detected in the GW population. The parents G122 and WMG904-20-3 represent Andean and *P*. *coccineus* sources of resistance, respectively. The latter was introgressed into a snap bean background using the backcross-inbred method with two backcrosses to the recurrent parent, OR91G, followed by three generations of single seed descent. The QTL on Pv03 detected for the WMG904-20-3 parent in the original backcross-inbred population [32] was not detected in this population. The cross between two Andean parents representing sources of resistance to white mold can make it more difficult to observe segregation for resistance and detect QTL. In the present case, the population showed a skewed distribution towards resistance which suggests that the two parents shared one or more resistance loci. Viteri et al. [80] observed only one gene conditioning resistance in G122/A195 population, where similar allelic similarities may be present.

The AO and GW genetic map distances are longer than for other maps. We attribute this to the GBS approach where a large set of SNPs were generated, and where imputation was used to estimate SNP allele calls for SNPs which were missing in individual lines. The imputation of missing data can cause false inflation of genetic distances [81].

It was surprising how robust the original RAPD- and AFLP-based maps (AN, R31, and XC) with relatively few markers were for detecting QTL conditioning partial resistance to white mold, as there were only a few QTL identified in the new linkage maps that were undetected by the original maps (Table 1). The WM7.5 QTL was detected in the new map for AN (Figs H and N in S1 File) albeit it has a low LOD value (2.64) and was derived from the susceptible parent. The opposite also occurred, as the originally mapped WM4.2 and WM8.4 were not detected, WM5.3 and WM5.4 merged into one QTL, and WM7.3 shifted to WM7.5 in the denser SNP map for R31 (Figs G and Q in S1 File). There were five QTL identified for straw test resistance in the original and new SNP maps for XC population. But based on physical positions from the SNP map, WM3.2 from the original XC map shifted position to WM3.1 and WM6.1 shifted to WM6.2.

## Conclusion

New genomic resources in common bean enabled us to compare 37 individual QTL conditioning partial resistance to white mold from across 14 RIL populations. Knowledge of the physical position and interval for the 37 QTL facilitated the identification of nine meta-QTL and verifies the complexity of the quantitative resistance response to *S*. *sclerotiorum* in common bean. Breeders have struggled using MAS for QTL conferring partial resistance in the past because sparse maps and unknown physical positions contributed to the selection of large genomic intervals leading to negative linkage drag for yield and other traits. Moreover, the derivation of the resistance from exotic sources and wide crosses such as Middle American x Andean, dry bean x snap bean, and cultivated x landrace or wild, contributes to negative linkage drag effects on yield and other traits. To overcome these constraints it is important to identify stable QTL by continuously validating and characterizing the effects of the individual QTL in more populations and environments, and by different methods. Verified meta-QTL should then be fine mapped using near-isogenic populations or other approaches to develop MAS for a smaller genomic interval, and to facilitate the search for the actual gene(s) underpinning the resistance. Viable candidate genes (cell wall receptor kinase, coronatine-insensitive protein 1—*COI1*, ethylene responsive transcription factor, peroxidase, and MYB transcription factor) with differential expression under *S*. *sclerotiorum* infection in other studies were found within the narrower CI for five of the nine meta-QTL identified in this study. Going forward, the nine meta-QTL provide targets for further characterization and manipulation by breeders. As new information is incorporated, the genomic confidence interval for the individual meta-QTL can be refined and their utility for breeding reassessed.

## Supporting information

S1 File **Genetic linkage maps (Figs A to I) and significant QTL depicted (Figs J to R).** Fig A: Genetic linkage maps Orion//Orion/R31-83 (O83), Fig B: Montrose/I9365-25 (M25), Fig C: UI-537/I9365-25 (U25), Fig D: Orion/USPT-WM-12 (O12), Fig E: A195/OSU6137 (AO) population, Fig F: G122/WMG904-20-3 (GW), Fig G: Raven/I9365-31 (R31), Fig H: Aztec/ND88-106-04 (AN), and Fig I: Xana/Cornell49-242 (XC) populations with cM distance left of chromosomes. Fig J: WM (white mold) resistance QTL detected in XC, Fig K: O83, Fig L: AO, Fig M: O12, Fig N: AN, Fig O: M25, Fig P: U25, Fig Q: R31, Fig R: GW populations. The highlighted markers represent cofactors. Horizontal solid line represents significance threshold of P < 0.05% based on 1000 permutations for the trait with the lowest LOD value.
(PDF)

S1 Table SNPs that define the start, peak, and end position of white mold meta-QTL in common bean. (XLSX)
