# Supplementary material for: Meta-QTL for resistance to white mold in common bean
Source: PLoS One. 2017 Feb 15;12(2):e0171685. doi: 10.1371/journal.pone.0171685 (PMC5310892; doi:10.1371/journal.pone.0171685)
Supplement: S1 File — Genetic linkage maps (Figs A to I) and significant QTL depicted (Figs J to R). Fig A: Genetic linkage maps Orion//Orion/R31-83 (O83), Fig B: Montrose/I9365-25 (M25), Fig C: UI-537/I9365-25 (U25), Fig D: Orion/USPT-WM-12 (O12), Fig E: A195/OSU6137 (AO) population, Fig F: G122/WMG904-20-3 (GW), Fig G: Raven/I9365-31 (R31), Fig H: Aztec/ND88-106-04 (AN), and Fig I: Xana/Cornell49-242 (XC) populations with cM distance left of chromosomes. Fig J: WM (white mold) resistance QTL detected in XC, Fig K: O83, Fig L: AO, Fig M: O12, Fig N: AN, Fig O: M25, Fig P: U25, Fig Q: R31, Fig R: GW populations. The highlighted markers represent cofactors. Horizontal solid line represents significance threshold of P < 0.05% based on 1000 permutations for the trait with the lowest LOD value. (PDF) [file pone.0171685.s001.pdf]

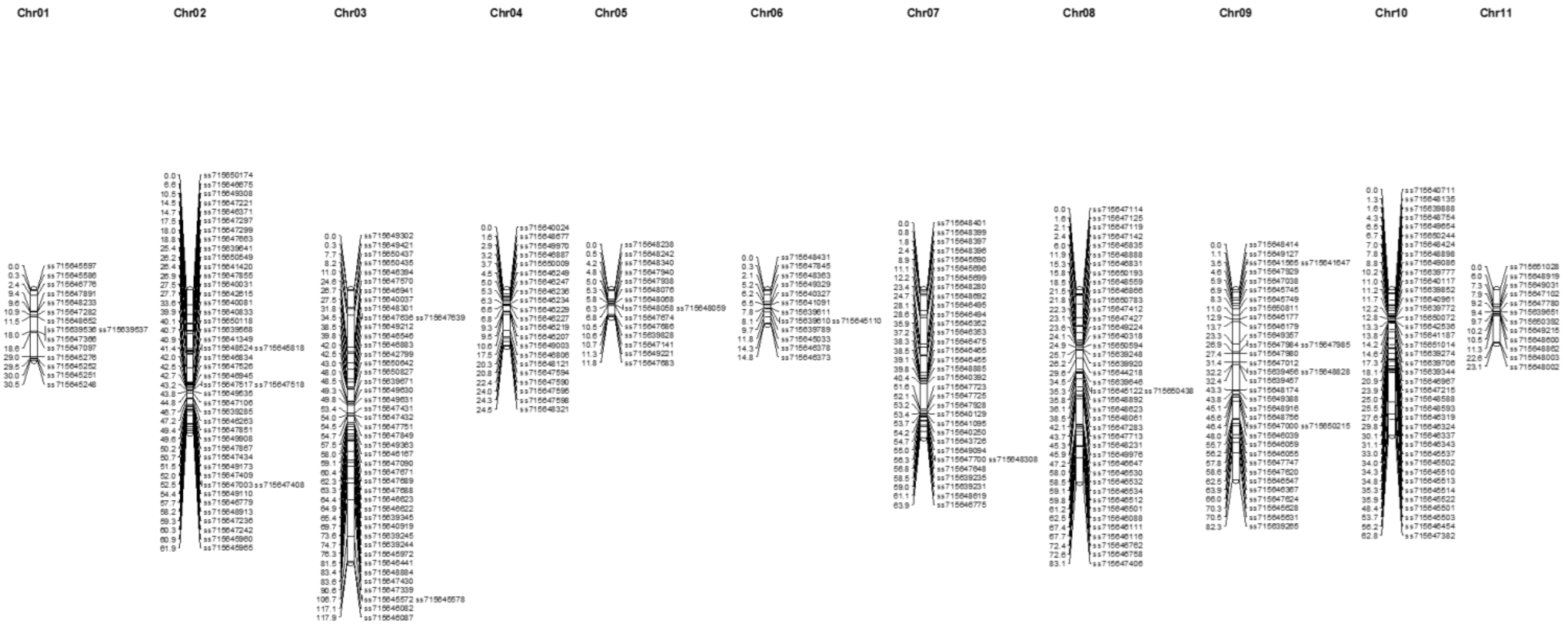

Fig A in S1 File. Genetic linkage map of the Orion//Orion/R31-83 (O83) RIL population. cM distance left of chromosomes.

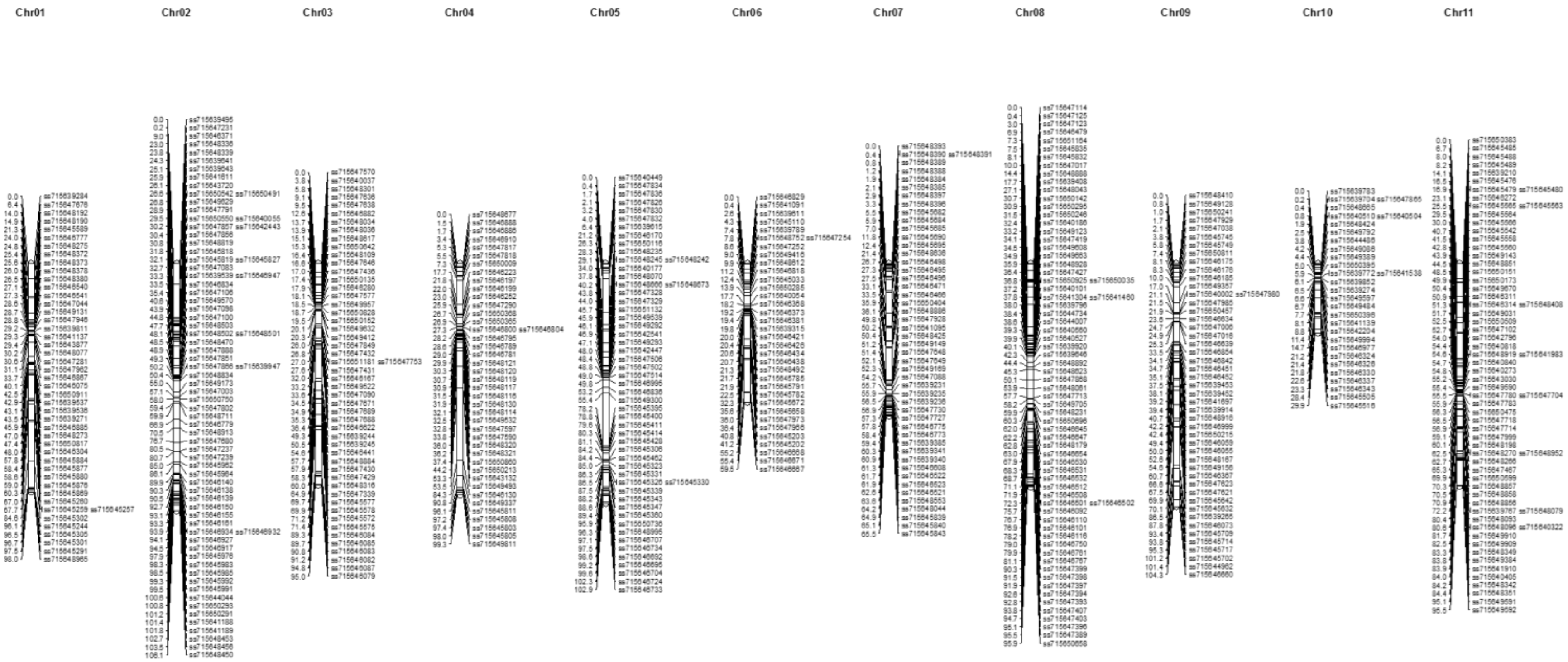

Fig B in S1 File. Genetic linkage map of the Montrose/I9365-25 (M25) population. cM distance left of chromosomes.

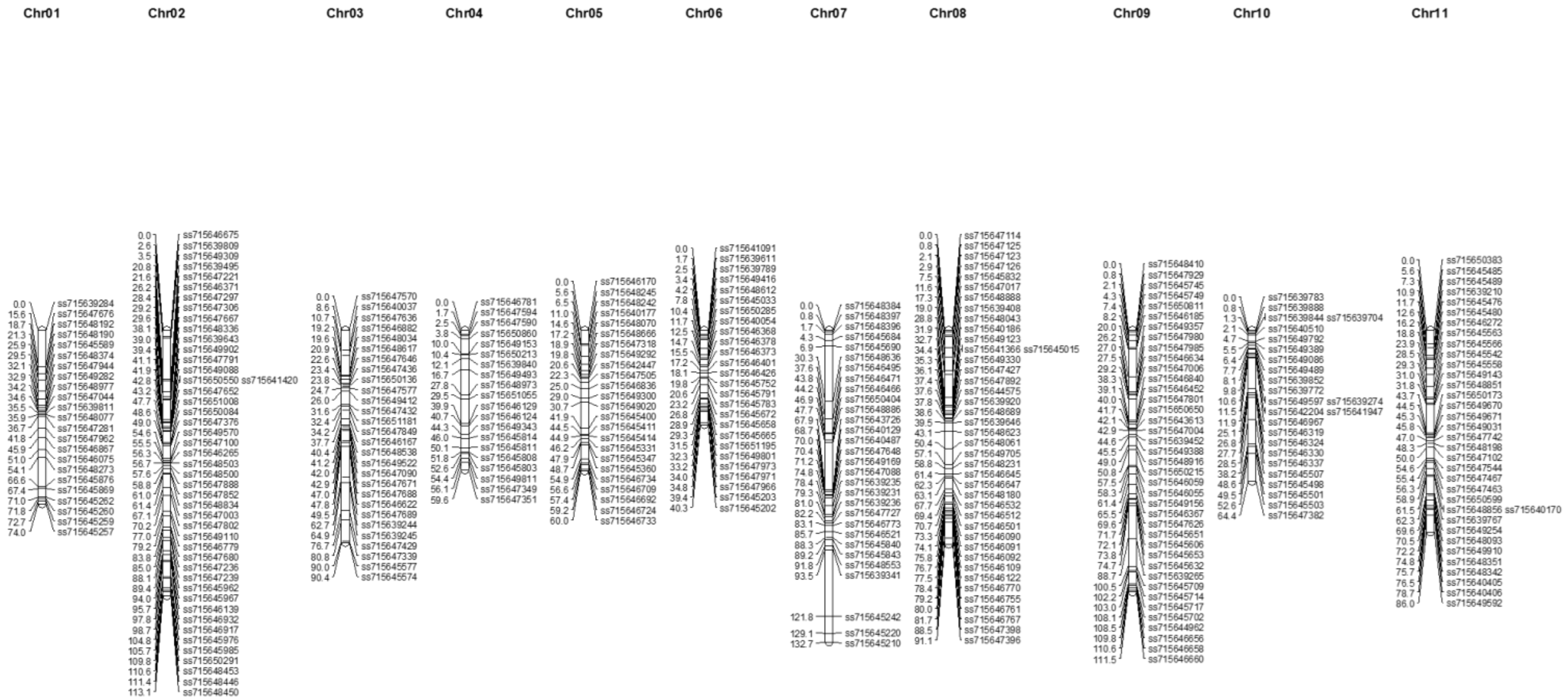

Fig C in S1 File. Genetic linkage map of the UI-537/I9365-25 (U25) population. cM distance left of chromosomes.

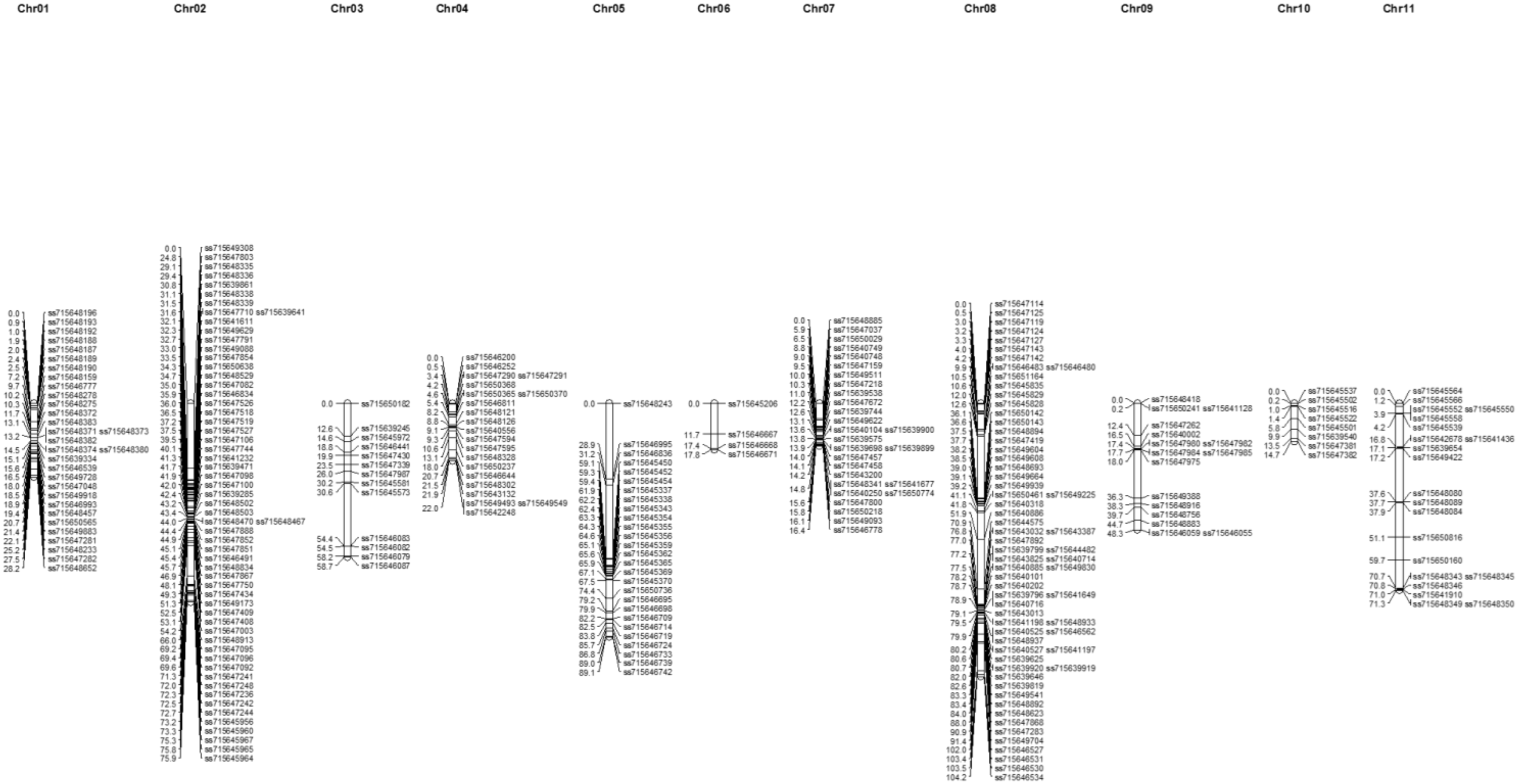

Fig D in S1 File. Genetic linkage map of the Orion/USPT-WM-12 (O12) population. cM distance left of chromosomes.

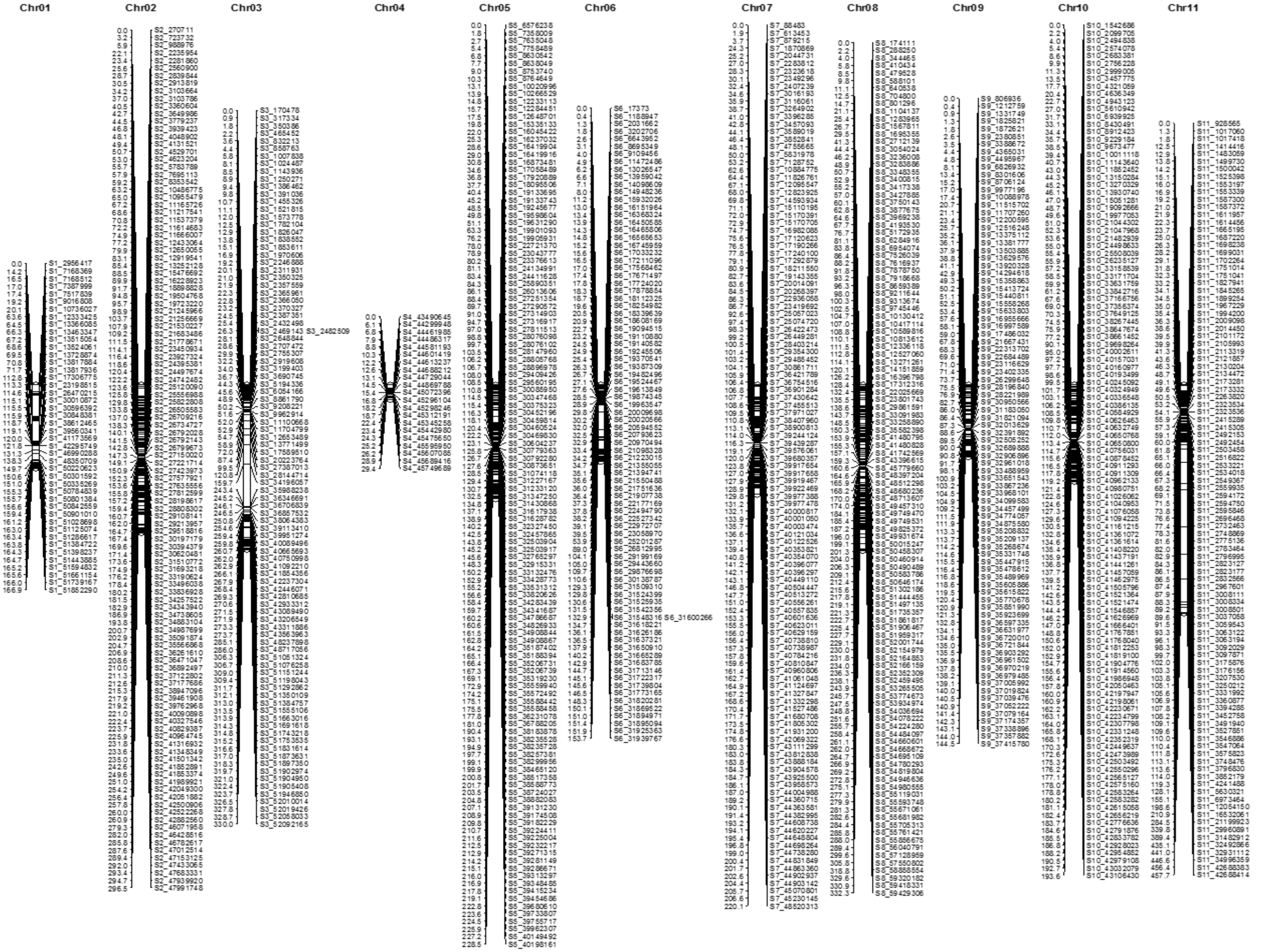

Fig E in S1 File. Genetic linkage map of the A195/OSU6137 (AO) population. cM distance left of chromosomes.

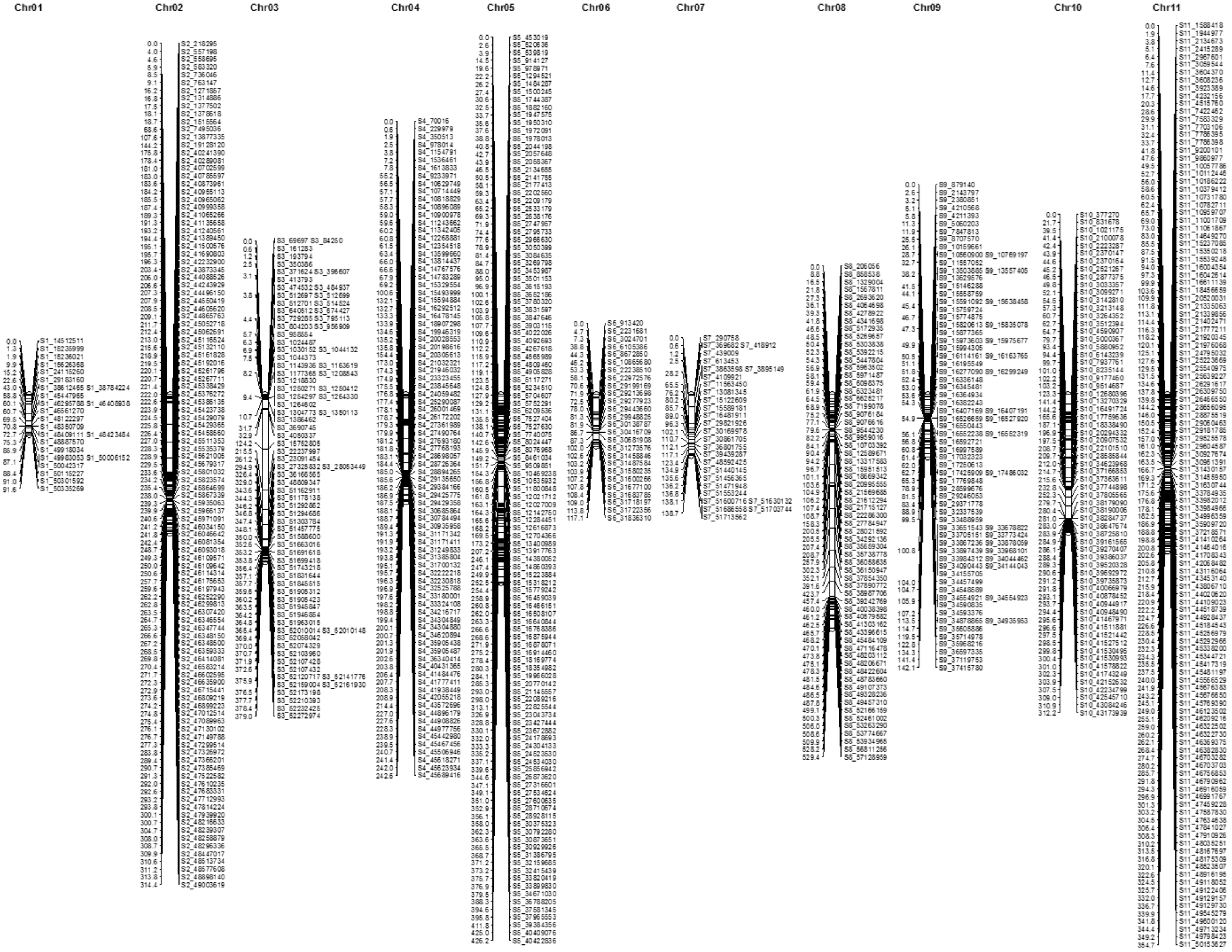

Fig F in S1 File. Genetic linkage map of the G122/WMG904-20-3 (GW) population. cM distance left of chromosomes.

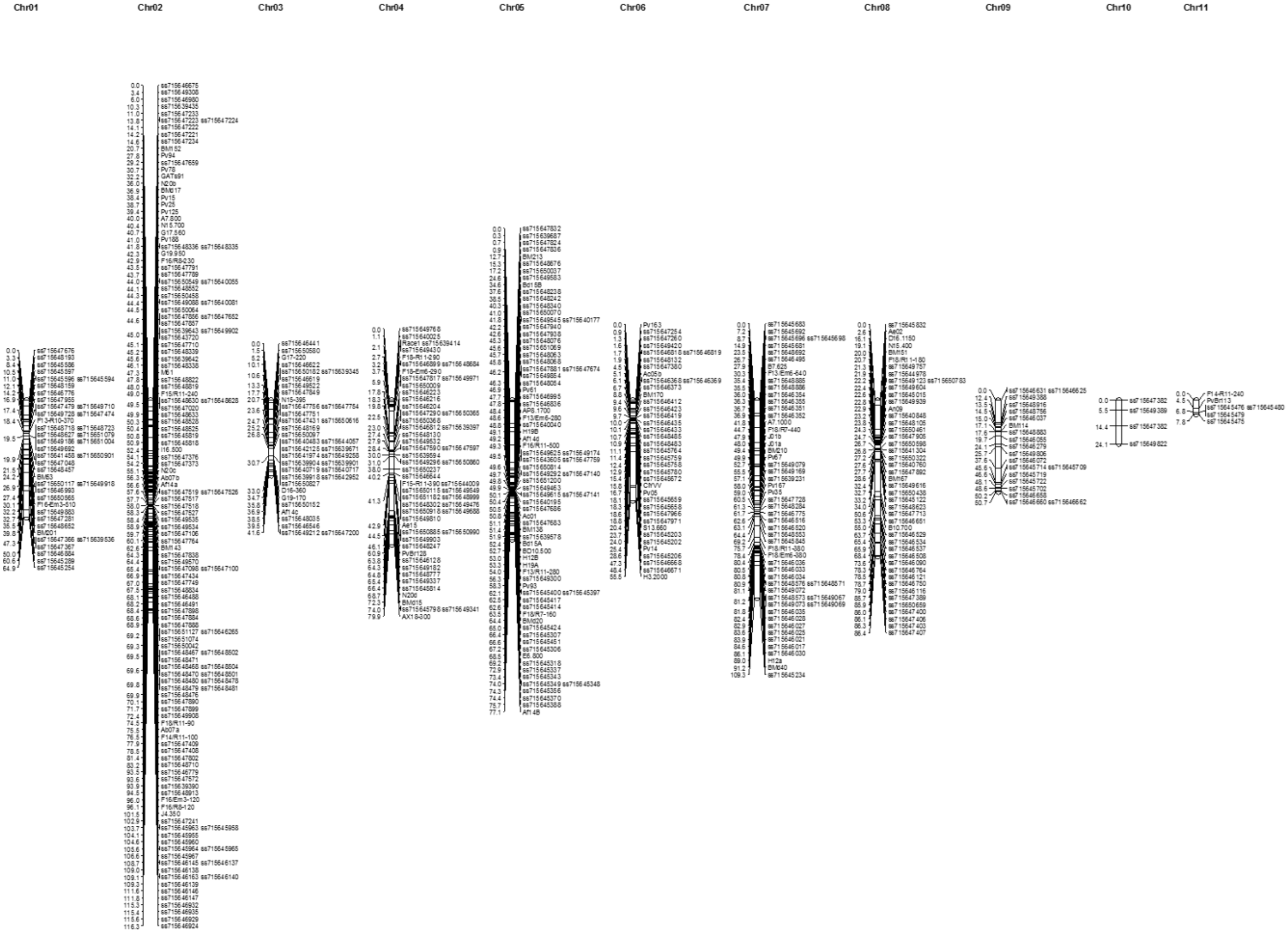

Fig G in S1 File. Genetic linkage map of the Raven/I9365-31 (R31) population. cM distance left of chromosomes.

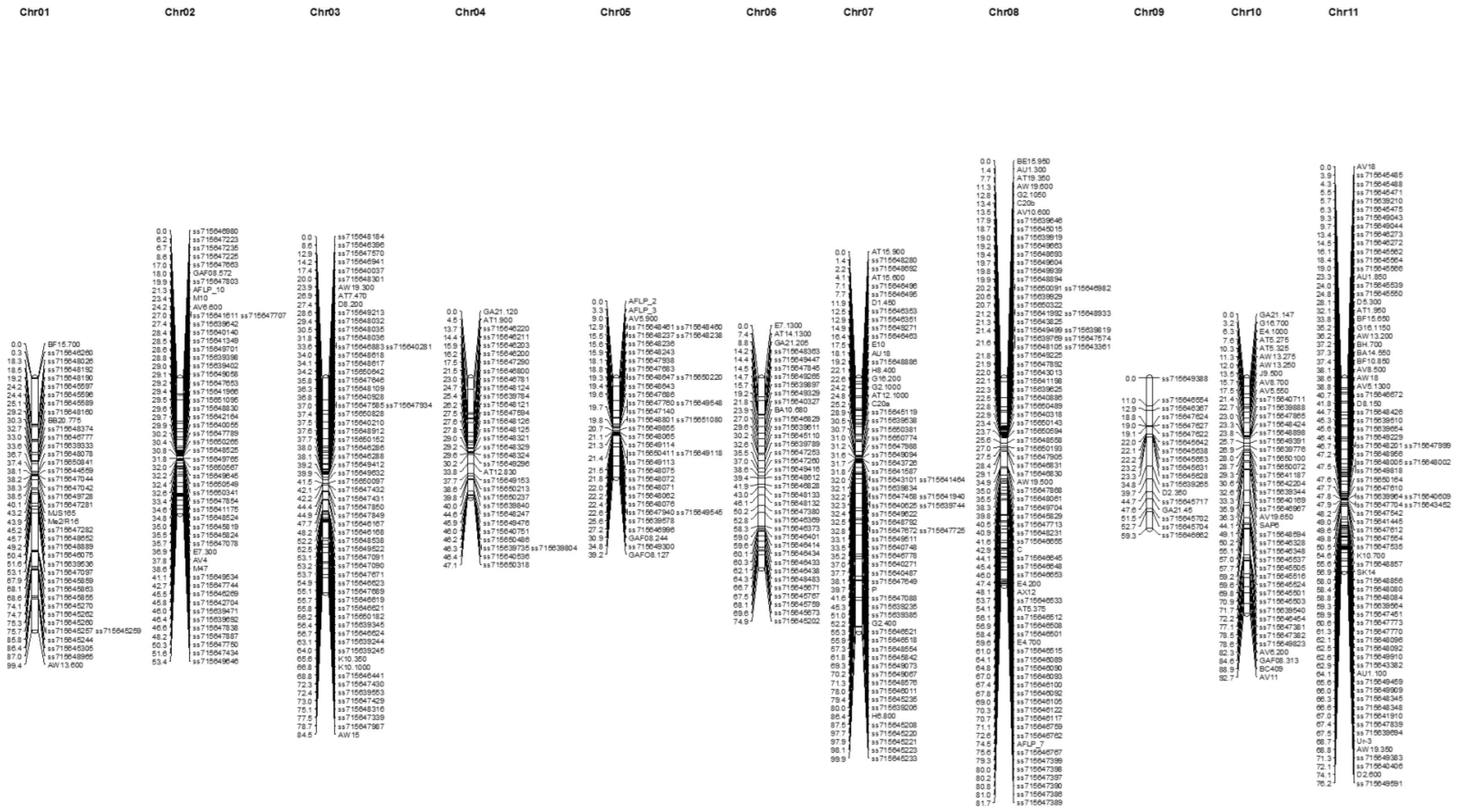

Fig H in S1 File. Genetic linkage map of the Aztec/ND88-106-04 (AN) population. cM distance left of chromosomes.

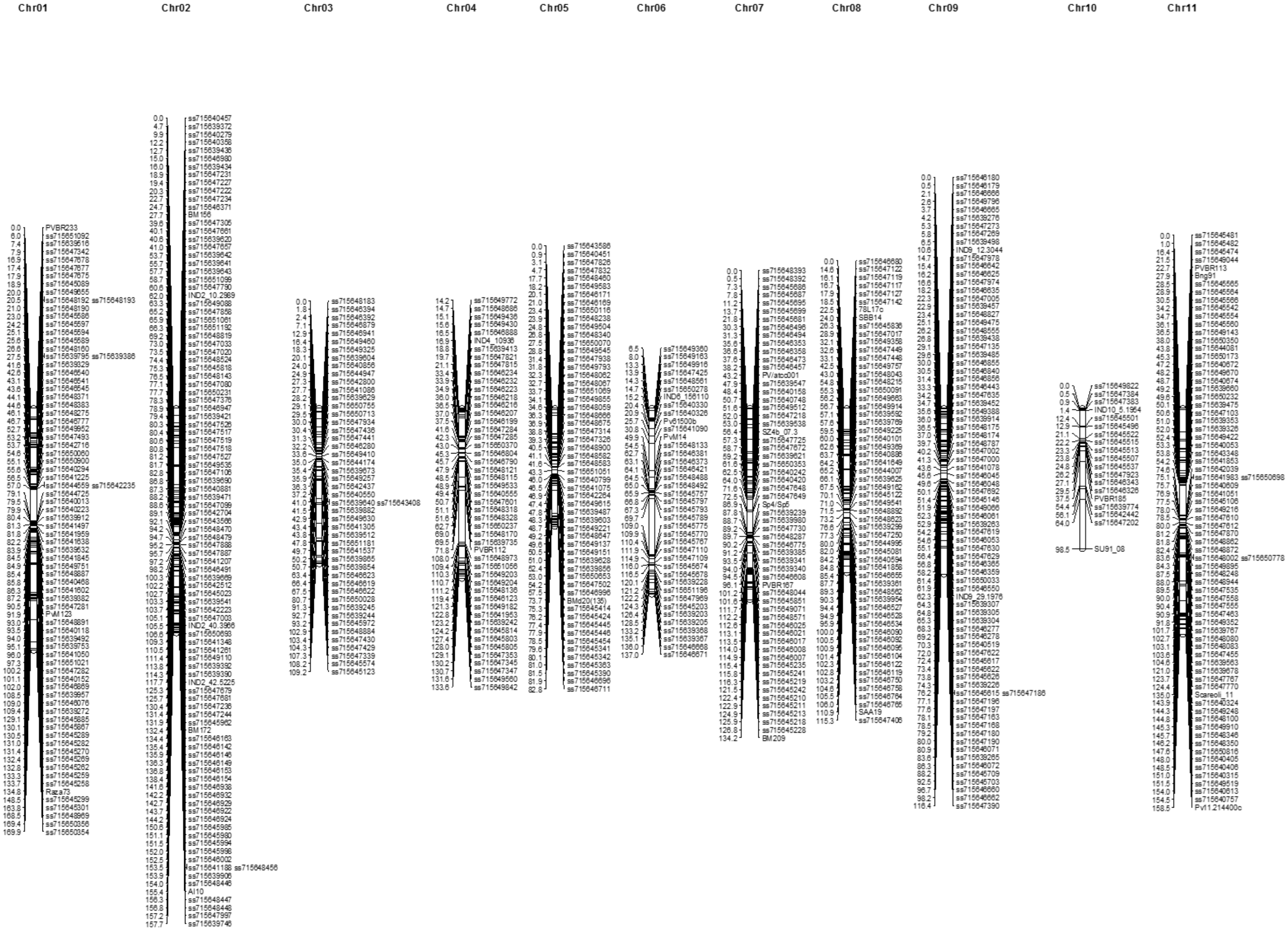

Fig I in S1 File. Genetic linkage map of the Xana/Cornell49-242 (XC) population. cM distance left of chromosomes.

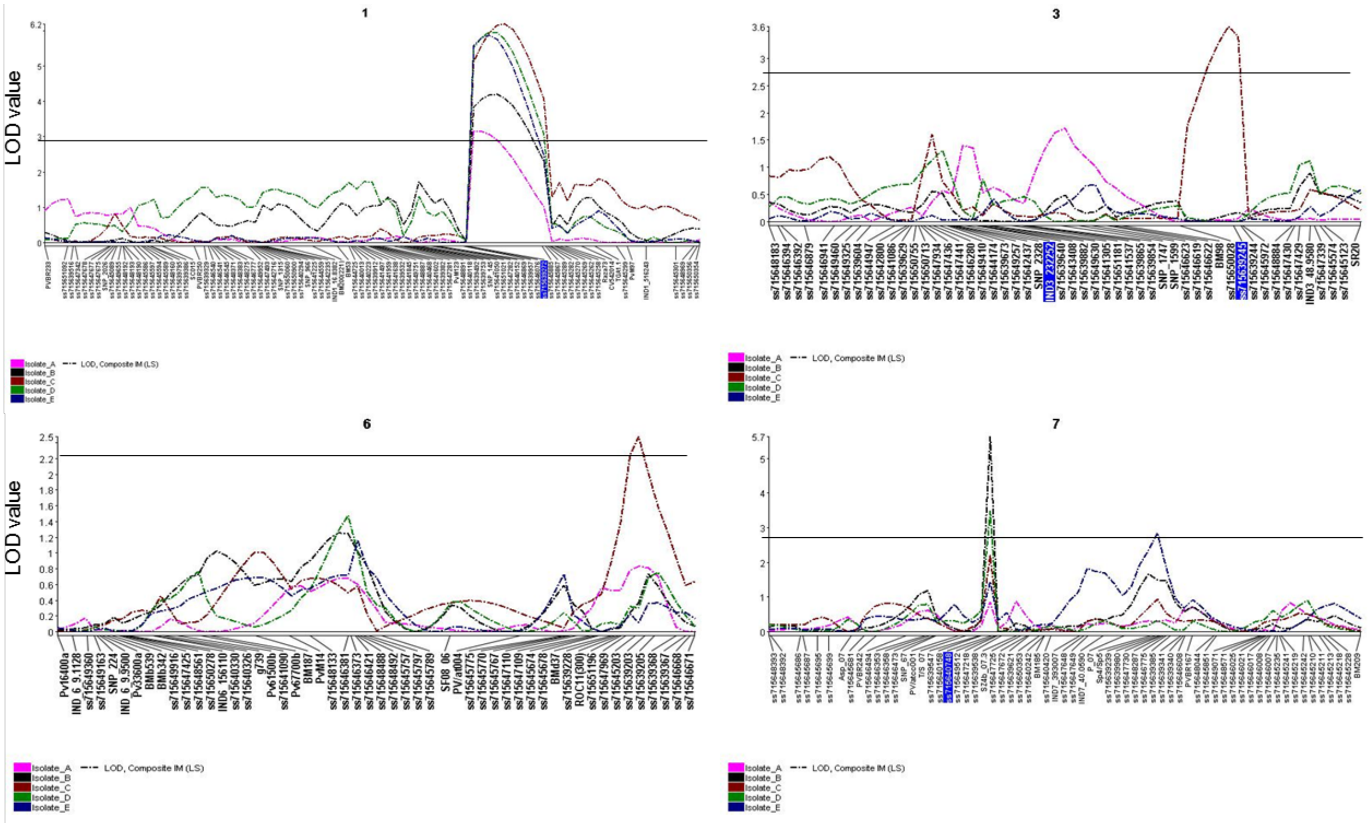

Fig J in S1 File. WM (white mold) resistance QTL detected in XC population. QTL on chromosomes 1 (WM1.1), 3 (WM3.1), 6 (WM6.2) and 7 (WM7.1 and WM7.4) associated with the means of the white mold scores against five different *S. sclerotiorum* isolates using the straw test method for the RIL population Xana/Cornell49-242 (XC) (See Pérez-Vega et al. [37] for details). The highlighted markers represent cofactors. Horizontal solid line represents significance threshold of P < 0.05% based on 1000 permutations for the trait with the lowest LOD value.

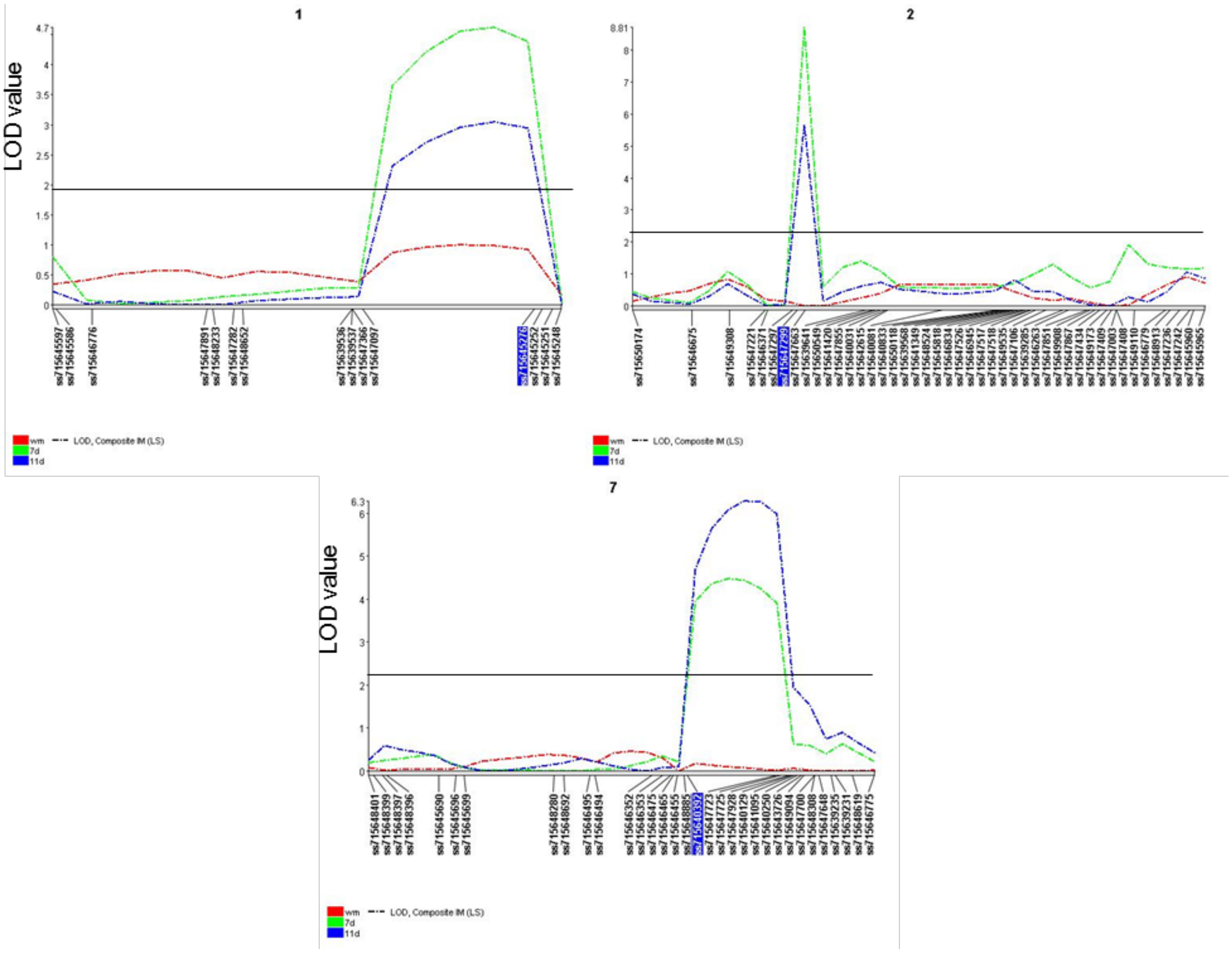

Fig K in S1 File. WM (white mold) resistance QTL detected in O83 population. QTL on chromosomes 1 (WM1.1), 2 (WM2.2) and 7 (WM7.6) associated with the means of the white mold scores using the straw test method for the advanced backcross population Orion//Orion/R31-83 (O83). wm = field, 7d and 11d represents straw test ratings days after inoculation. The highlighted markers represent cofactors. Horizontal solid line represents significance threshold of P < 0.05% based on 1000 permutations for the trait with the lowest LOD value.

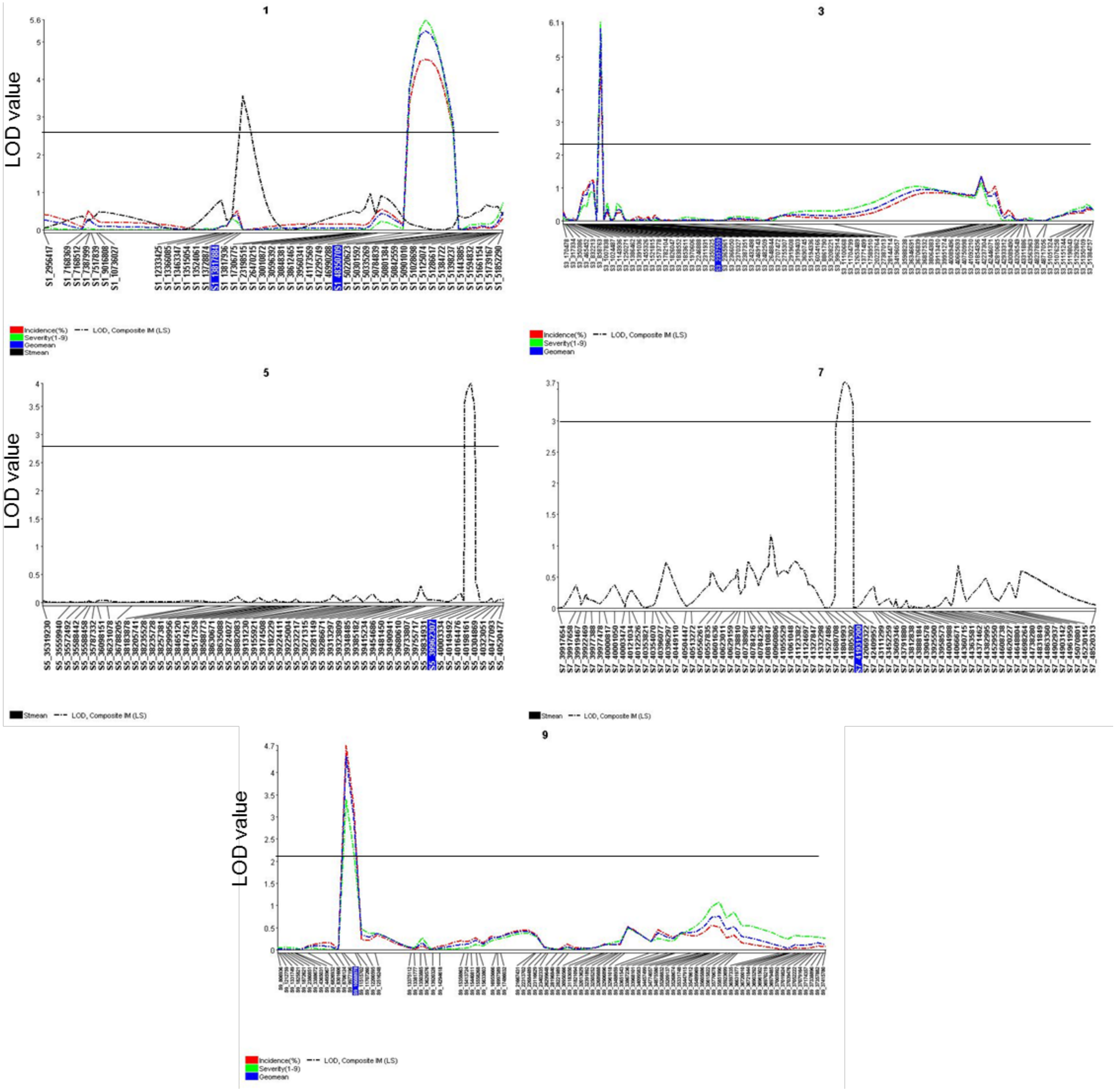

Fig L in S1 File. WM (white mold) resistance QTL detected in AO population. QTL on chromosomes 1 (WM1.1 and WM1.3), 3 (WM3.2), 5 (WM5,5), 7 (WM7.4) and 9 (WM9.3) associated with the means of the white mold scores evaluated in the field for the RIL population A195/OSU6137 (AO). Stmean = mean of straw test scores in the greenhouse Incidence (%) = percentage of infected plants in a plot. Severity (1-9) = disease rate, where 1 indicate no disease and 9 indicate that all plants were dead. Geomean = geometric mean calculated based in the incidence percentage and severity scores. The highlighted markers represent cofactors. Horizontal solid line represents significance threshold of P < 0.05% based on 1000 permutations for the trait with the lowest LOD value.

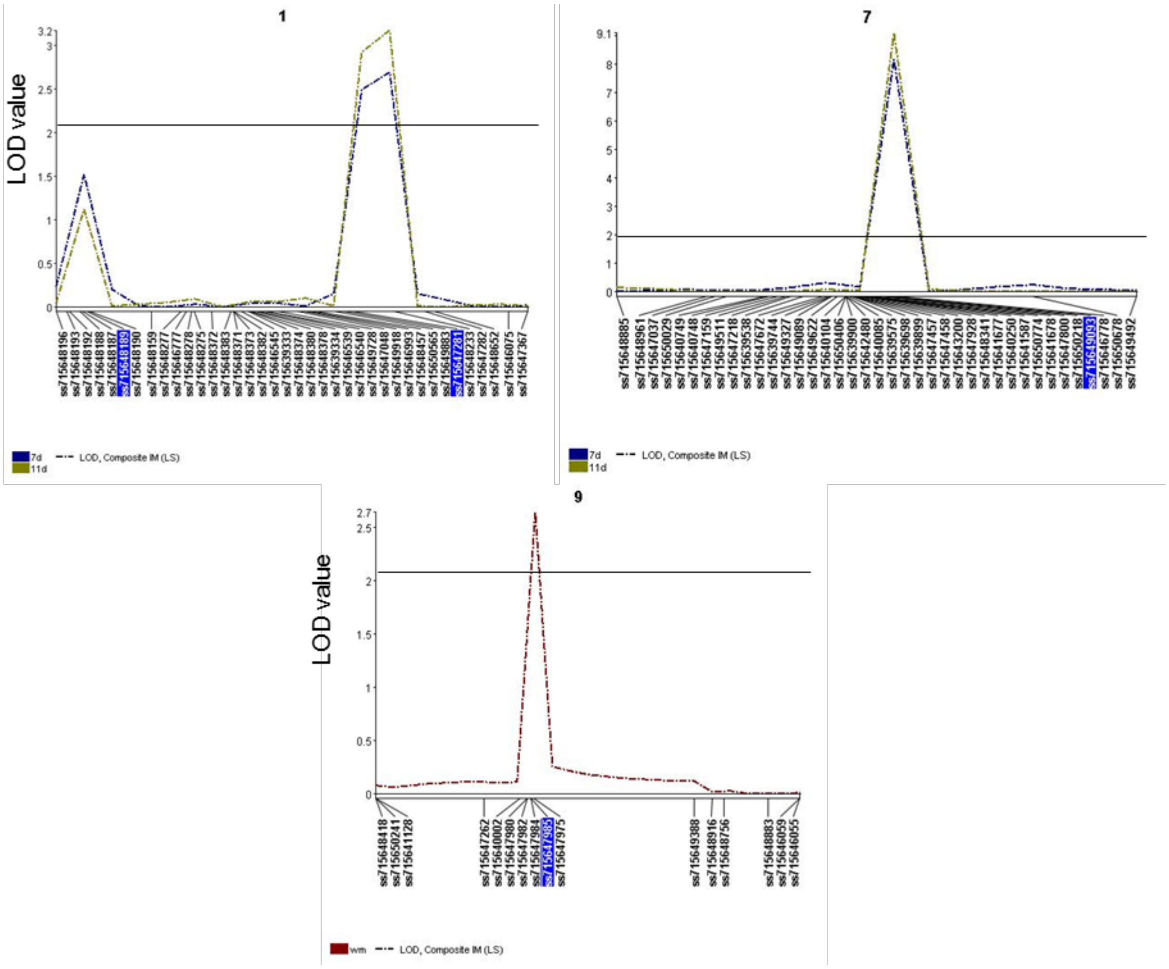

Fig M in S1 File. WM (white mold) resistance QTL detected in O12 population. QTL on chromosomes 1 (WM1.2), 7 (WM7.4) and 9 (WM9.3) associated with the means of the white mold scores using the straw test method in the greenhouse and evaluated in the field for the RIL population Orion/USPT-WM-12 (O12). wm = field, 7d and 11d represents straw test ratings days after inoculation. The highlighted markers represent cofactors. Horizontal solid line represents significance threshold of P < 0.05% based on 1000 permutations of the trait with the lowest LOD value.

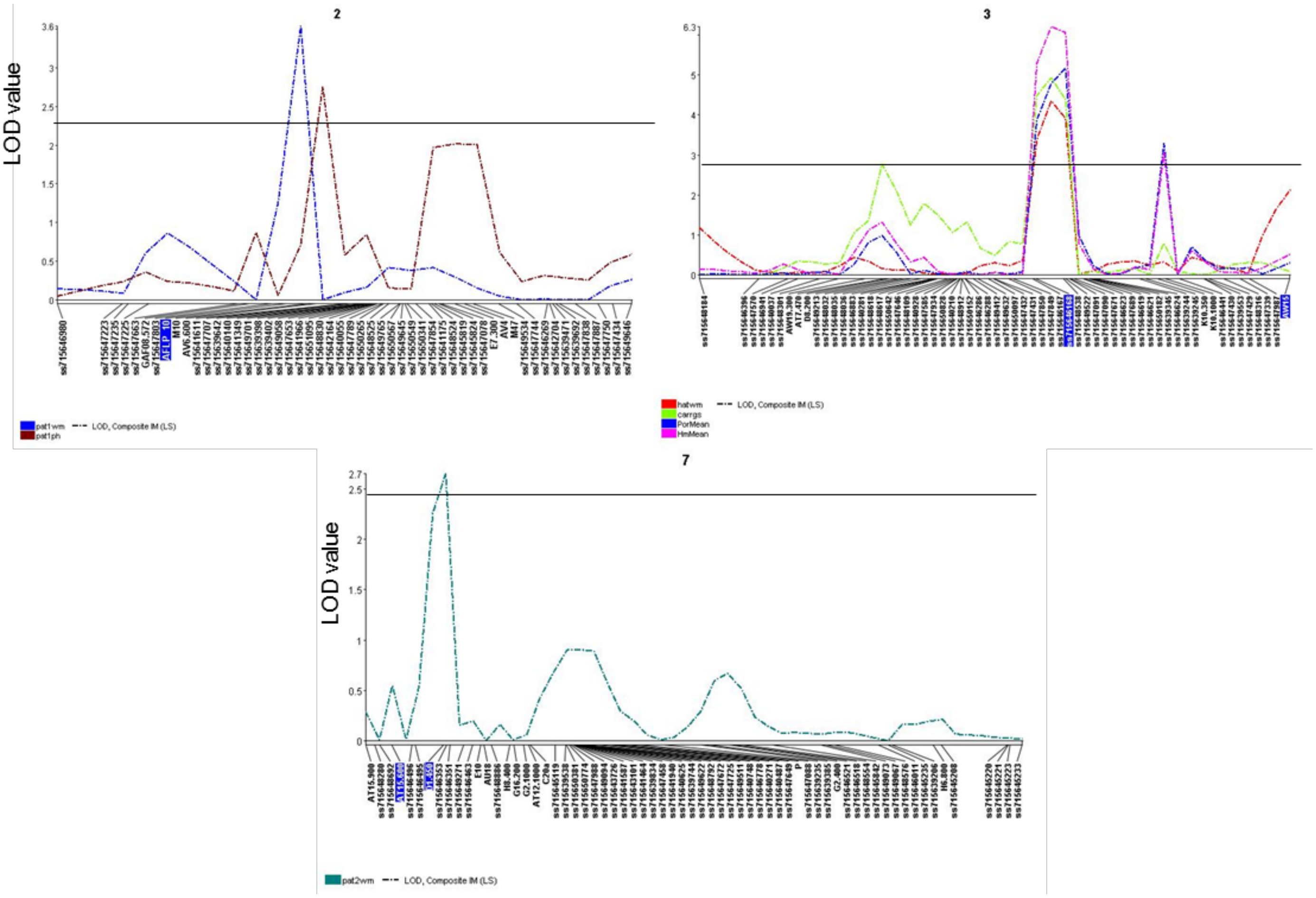

Fig N in S1 File. WM (white mold) resistance QTL detected in AN population. QTL on chromosomes 2 (WM2.2), 3 (WM3.1) and 7 (WM7.5) associated with the means of the white mold scores in the field for the RIL population Aztec/ND88-106-04 (AN). pat1, pat2, carr and hat and refers to different field environment. wm = white mold disease score, ph = plant height, gs = green stem, PorMean = canopy porosity mean of all environments, HmMean = harvest maturity mean of all environments (See Miklas et al. [15] for details). The highlighted markers represent cofactors. Horizontal solid line represents significance threshold of P < 0.05% based on 1000 permutations for the white mold trait with the lowest LOD value.

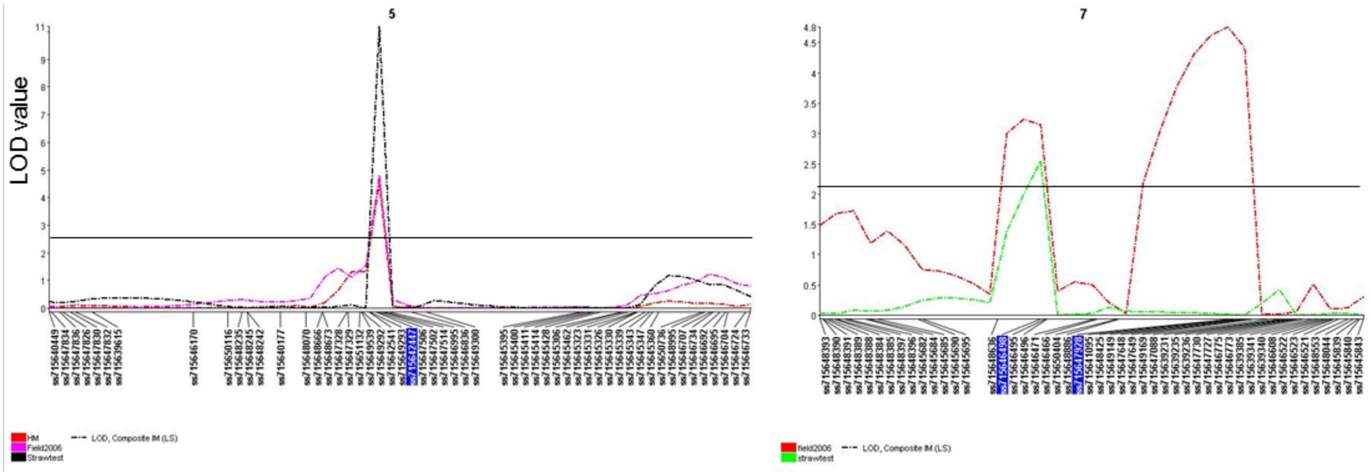

Fig O in S1 File. WM (white mold) resistance QTL detected in M25 population. QTL on chromosomes 5 (WM5.4) and 7 (WM7.1 – field and WM7.5 – field and straw test) associated with the means of the white mold scores using the straw test method in the greenhouse and evaluated in the field for the RIL population Montrose/I9365-25 (M25). HM = harvest maturity. The highlighted markers represent cofactors. Horizontal solid line represents significance threshold of $P < 0.05\%$ based on 1000 permutations for the white mold trait with the lowest LOD value.

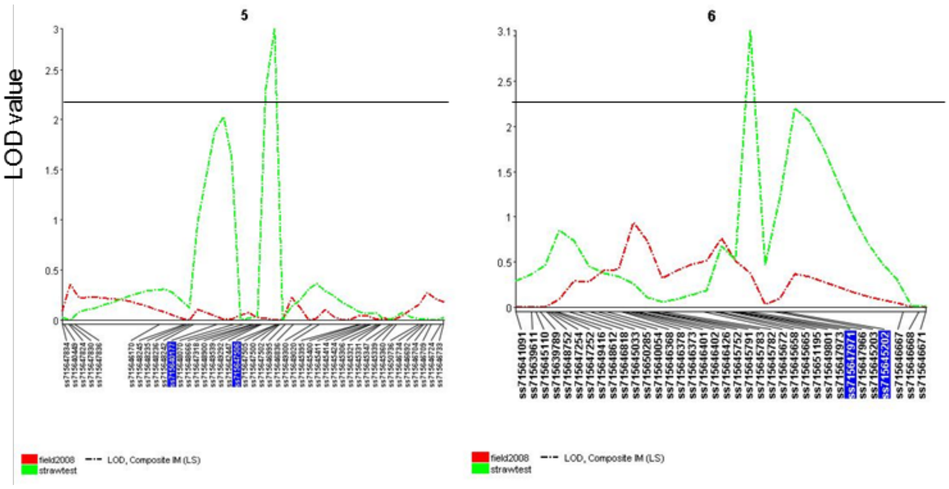

Fig P in S1 File. WM (white mold) resistance QTL detected in U25 population. QTL on chromosomes 5 (WM5.4) and 6 (WM6.2) associated with the means of the white mold scores using the straw test method in the greenhouse and evaluated in the field for the RIL population UI-537/I9365-25 (U25). The highlighted markers represent cofactors. Horizontal solid line represents significance threshold of P < 0.05% based on 1000 permutations.

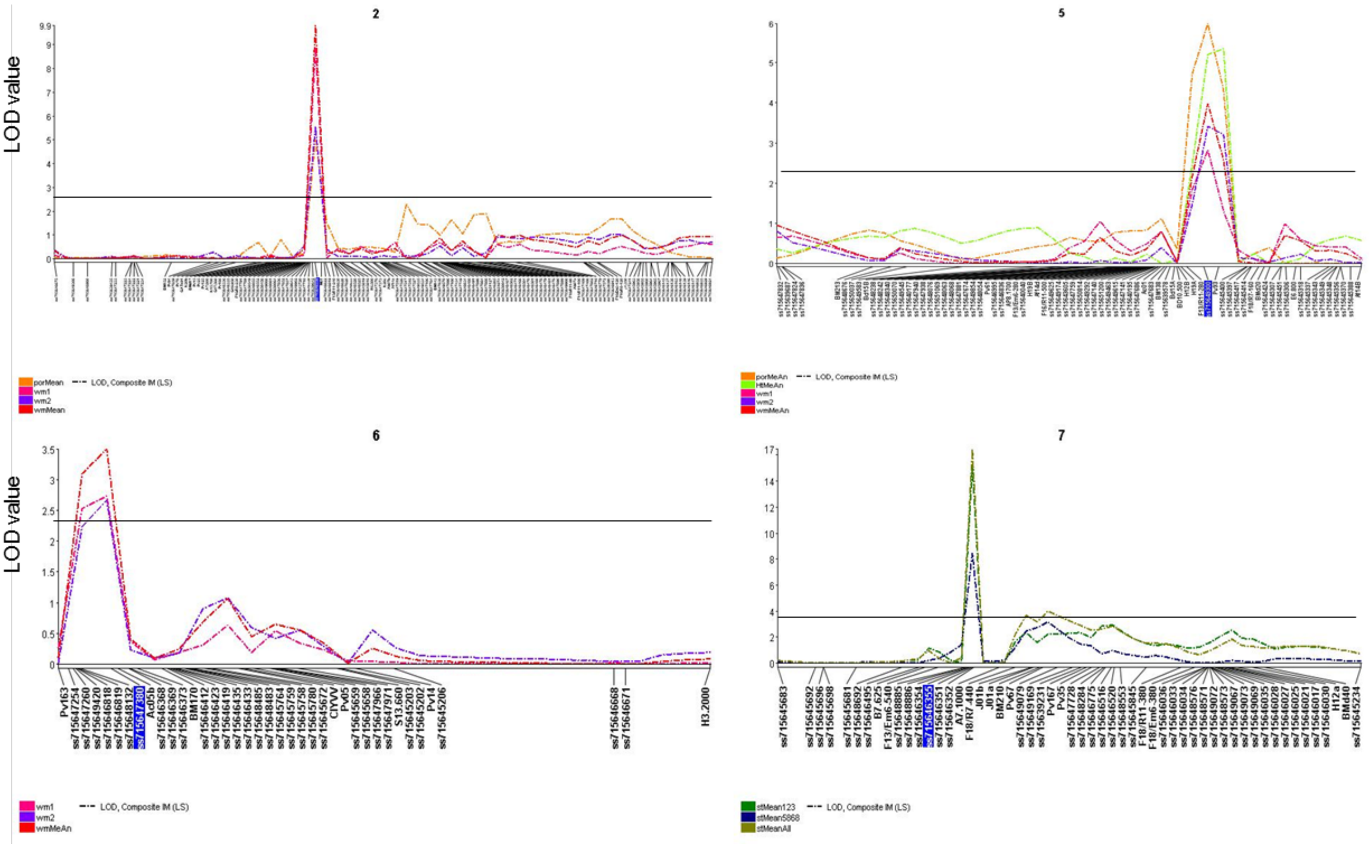

Fig Q in S1 File. WM (white mold) resistance QTL detected in R31 population. QTL on chromosomes 2 (WM2.2), 5 (WM5.4), 6 (WM6.1) and 7 (WM7.5) associated with the means of the white mold scores using the straw test method in the greenhouse and evaluated in the field for the RIL population Raven/I9365-31 (R31). wm1 and wm2 = different field environments, stMean123 represent straw results across three tests and stMean5868 = a separate straw test conducted at a cooler 58F/68F night/day temperature, porMean = canopy porosity mean of all field environments, HtMean = Canopy height mean for all field environments (See Soule et al. [5] for details). The highlighted markers represent cofactors. Horizontal solid line represents significance threshold of P < 0.05% based on 1000 permutations for the white mold trait with the lowest LOD value.

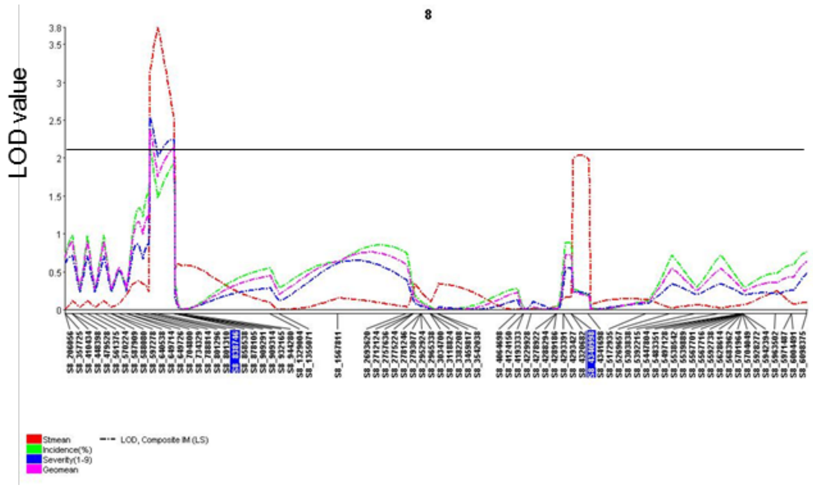

Fig R in S1 File. WM (white mold) resistance QTL detected in GW population. QTL on chromosome 8 (WM8.1) associated with the means of the white mold scores for the RIL population G122/WMG904-20-3 (GW). Partial linkage group for the chromosome depicted. Stmean = mean of straw test scores in the greenhouse. Incidence (%) = percentage of infected plants in a field plot. Severity (1-9) = field disease rating, where 1 indicate no disease and 9 indicate that all plants were dead. Geomean = geometric mean calculated based on the incidence and severity scores. The highlighted markers represent cofactors. Horizontal solid line represents significance threshold of P < 0.05% based on 1000 permutations for the trait with the lowest LOD value.
